# Supplementary material for: Identification of a Novel Class of Anti-Melanogenic Compounds, (Z)-5-(Substituted benzylidene)-3-phenyl-2-thioxothiazolidin-4-one Derivatives, and Their Reactive Oxygen Species Scavenging Activities
Source: Antioxidants (Basel). 2022 May 11;11(5):948. doi: 10.3390/antiox11050948 (PMC9137581; doi:10.3390/antiox11050948)
Supplement: Supplementary file 1 [file antioxidants-11-00948-s001.zip › antioxidants-1714436-supplementary.pdf]

Supplementary Materials
For

Identification of a novel class of anti-melanogenic compounds, (*Z*)-5-(substituted benzylidene)-3-phenyl-2-thioxothiazolidin-4-one derivatives and reactive oxygen species scavenging activities

Yeongmu Jeong [a,1], Sojeong Hong [a,1], Hee Jin Jung [b,1], Sultan Ullah [c], YeJi Hwang [a], Heejeong Choi [a], Jeongin Ko [a], Jieun Lee [a], Pusoon Chun [d], Hae Young Chung [b], Hyung Ryong Moon [a,*]

[a]*Department of Manufacturing Pharmacy, College of Pharmacy, Pusan National University, Busan 46241, South Korea*

[b]*Department of Pharmacy, College of Pharmacy, Pusan National University, Busan 46241, South Korea*

[c]*Department of Molecular Medicine, The Scripps Research Institute, Florida 33458, USA*

[d]*College of Pharmacy and Inje Institute of Pharmaceutical Sciences and Research, Inje University, Gimhae, Gyeongnam 50834, South Korea*

Contents

Figure S1. ^{1}H NMR spectrum of compound **1**……….……………5

Figure S2. ^{13}C NMR spectrum of compound **1**………..…………………..…..…………………6

Figure S3. LRMS spectrum of compound **1**…..……………………..………………….....……7

Figure S3. HRMS spectrum of compound **1**…..……………………..………………………..8

Figure S5. ^{1}H NMR spectrum of compound **2**……..……………………………….………9

Figure S6. ^{13}C NMR spectrum of compound **2**……..………………….....…...………….………10

Figure S7. LRMS spectrum of compound **2**…………………………...………...…..……….11

Figure S8. HRMS spectrum of compound **2**……………………..……………………………12

Figure S9. ^{1}H NMR spectrum of compound **3**………..………………………………………….13

Figure S10. ^{13}C NMR spectrum of compound **3**………..…..…………………...…...……………….14

Figure S11. Proton-coupled ^{13}C NMR spectrum of compound **3**……………………………….15

Figure S12. LRMS spectrum of compound **3**…..……..………………..………………………..16

Figure S13. ^{1}H NMR spectrum of compound **4**…..…………..…………………………………17

Figure S14. ^{13}C NMR spectrum of compound **4**……………..…………………......…………….…18

Figure S15. LRMS spectrum of compound **4**………………..………………...………………...19

Figure S16. HRMS spectrum of compound **4**…..……………………..………………………..20

Figure S17. ^{1}H NMR spectrum of compound **5**………..………………………………....…..…..…..21

Figure S18. ^{13}C NMR spectrum of compound **5**……..……………………......………..………..22

Figure S19. LRMS spectrum of compound **5**……..…………………………………………23

Figure S20. HRMS spectrum of compound **5**…..……………………..……………………...…24

Figure S21. ^{1}H NMR spectrum of compound **6**……..………………………………...………....25

Figure S22. ^{13}C NMR spectrum of compound **6**……….....……………………………………..…26

Figure S23. LRMS spectrum of compound **6**………...………...…...……..………………....…27

Figure S24. HRMS spectrum of compound **6**……………………………………………………..28

Figure S25. ^{1}H NMR spectrum of compound **7**……………………………………………….29

Figure S26. ^{13}C NMR spectrum of compound **7**……………………………………………….30

Figure S27. LRMS spectrum of compound **7**…………………………………………………...31

Figure S28. HRMS spectrum of compound **7**……………………………………………………32

Figure S29. ^{1}H NMR spectrum of compound **8**……………………………………………….33

Figure S30. ^{13}C NMR spectrum of compound **8**……………………………………………..34

Figure S31. LRMS spectrum of compound **8**……………………………………………………35

Figure S32. HRMS spectrum of compound **8**…………………………………………………..36

Figure S33. ^{1}H NMR spectrum of compound **9**……………………………………………….37

Figure S34. ^{13}C NMR spectrum of compound **9**……………………………………………….38

Figure S35. LRMS spectrum of compound **9**…………………………………………………...39

Figure S36. HRMS spectrum of compound **9**………………………………………………...40

Figure S37. ^{1}H NMR spectrum of compound **10**……………………………………………..41

Figure S38. ^{13}C NMR spectrum of compound **10**…………………………………………….42

Figure S39. LRMS spectrum of compound **10**………………………………………………...43

Figure S40. HRMS spectrum of compound **10**……………………………………………….44

Figure S41. ^{1}H NMR spectrum of compound **11**……………………………………………..45

Figure S42. ^{13}C NMR spectrum of compound **11**…………………………………………...46

Figure S43. LRMS spectrum of compound **11**…………………………………………………47

Figure S44. HRMS spectrum of compound **11**……………………………………………….48

Figure S45. ^{1}H NMR spectrum of compound **12**……………………………………………..49

Figure S46. ^{13}C NMR spectrum of compound **12**…………………………………………….50

Figure S47. LRMS spectrum of compound **12**………………………………………………...51

Figure S48. HRMS spectrum of compound **12**…..……………………..……………………52

Figure S49. ^{1}H NMR spectrum of compound **13**....…………………………………...………...53

Figure S50. ^{13}C NMR spectrum of compound **13**……....…………………………………………54

Figure S51. LRMS spectrum of compound **13**……………….……………….………………..55

Figure S52. ^{1}H NMR spectrum of compound **14**..56

Figure S53. ^{13}C NMR spectrum of compound **14**…………………………………………..……57

Figure S54. LRMS spectrum of compound **14**…………………………..……………..………58

Figure S55. Primer sets used for qRT-PCR……………………………………………………59

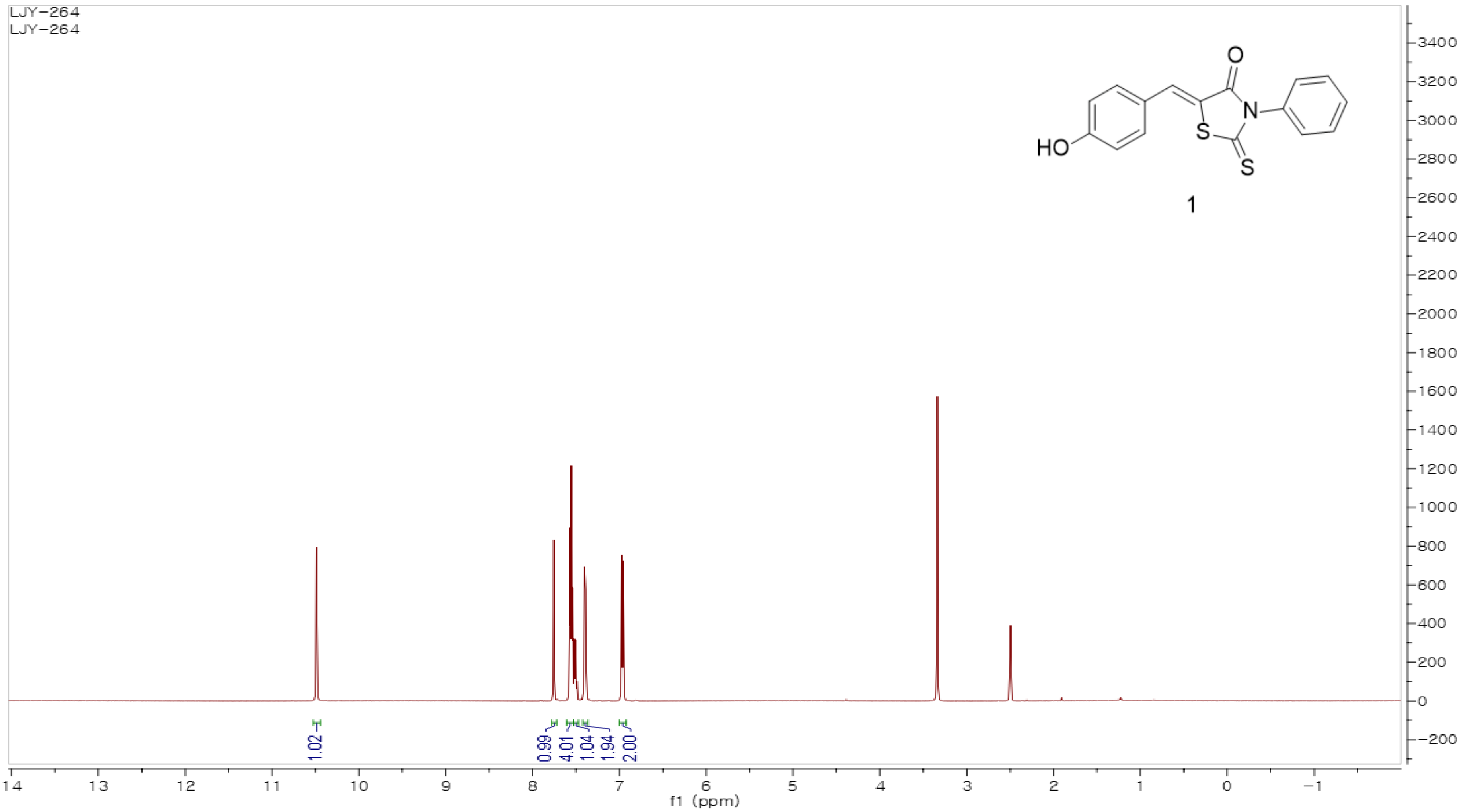

Figure S1. ^{1}H NMR spectrum of compound **1**

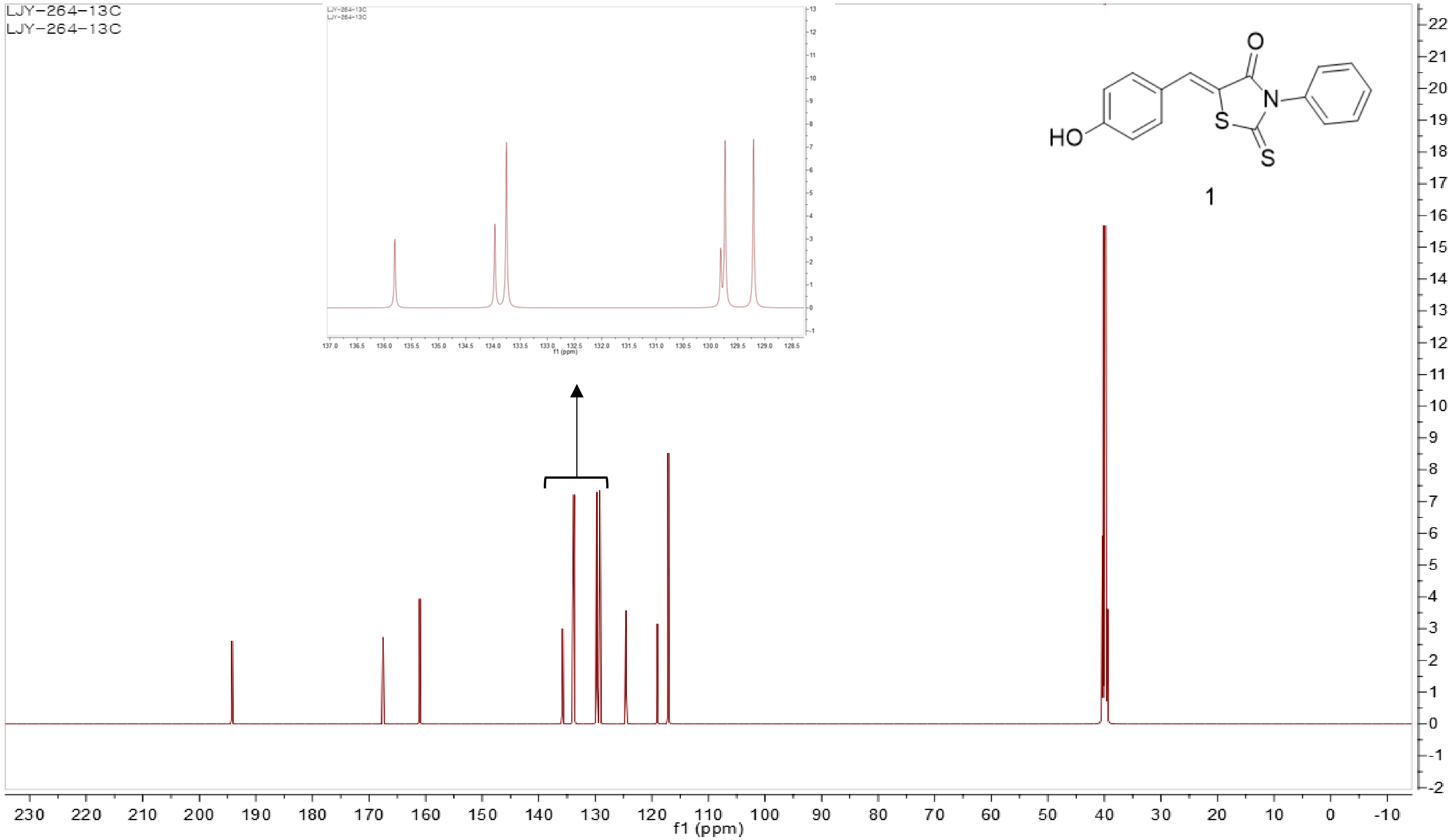

Figure S2. ^{13}C NMR spectrum of compound **1**

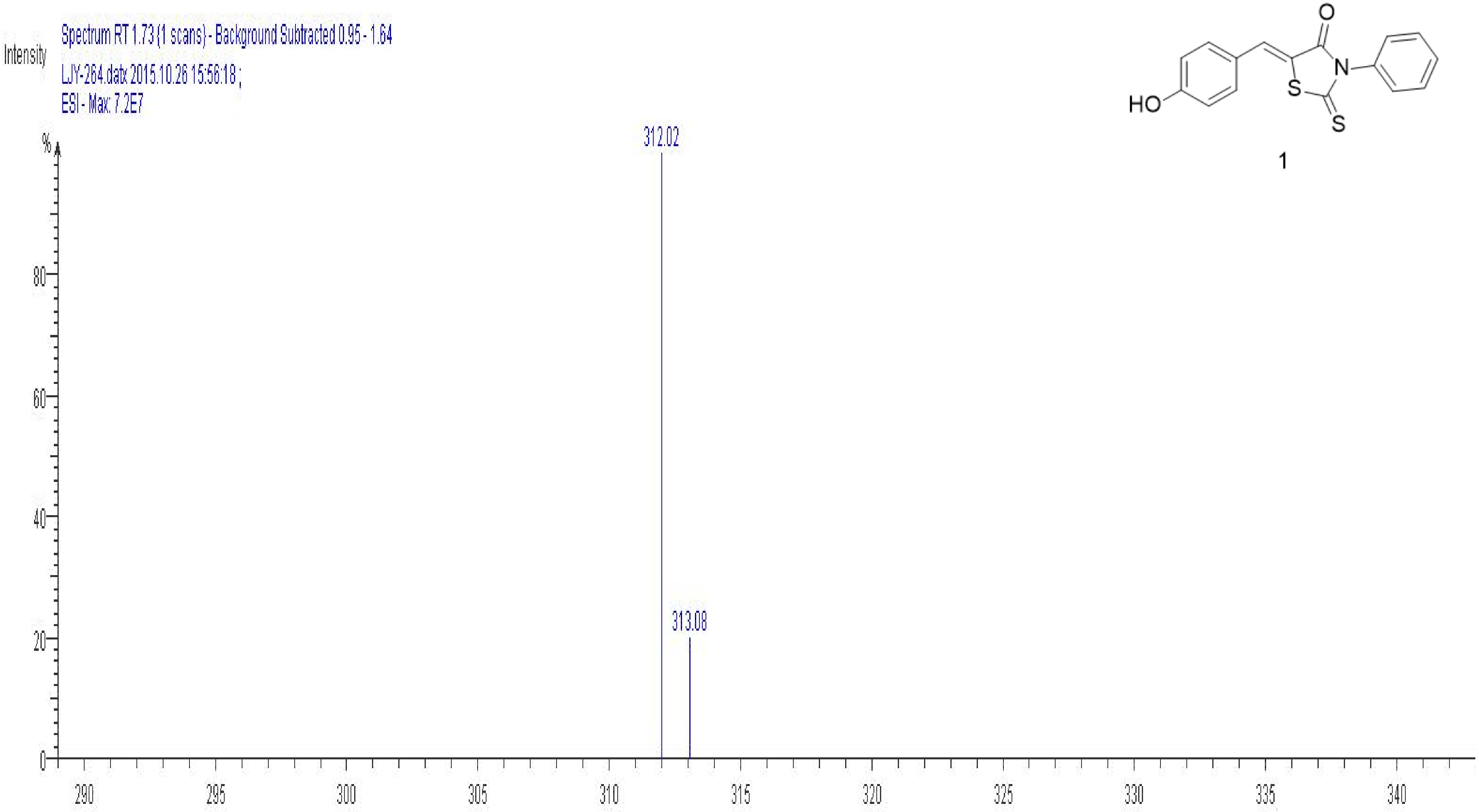

1

Figure S3. LRMS spectrum of compound **1**

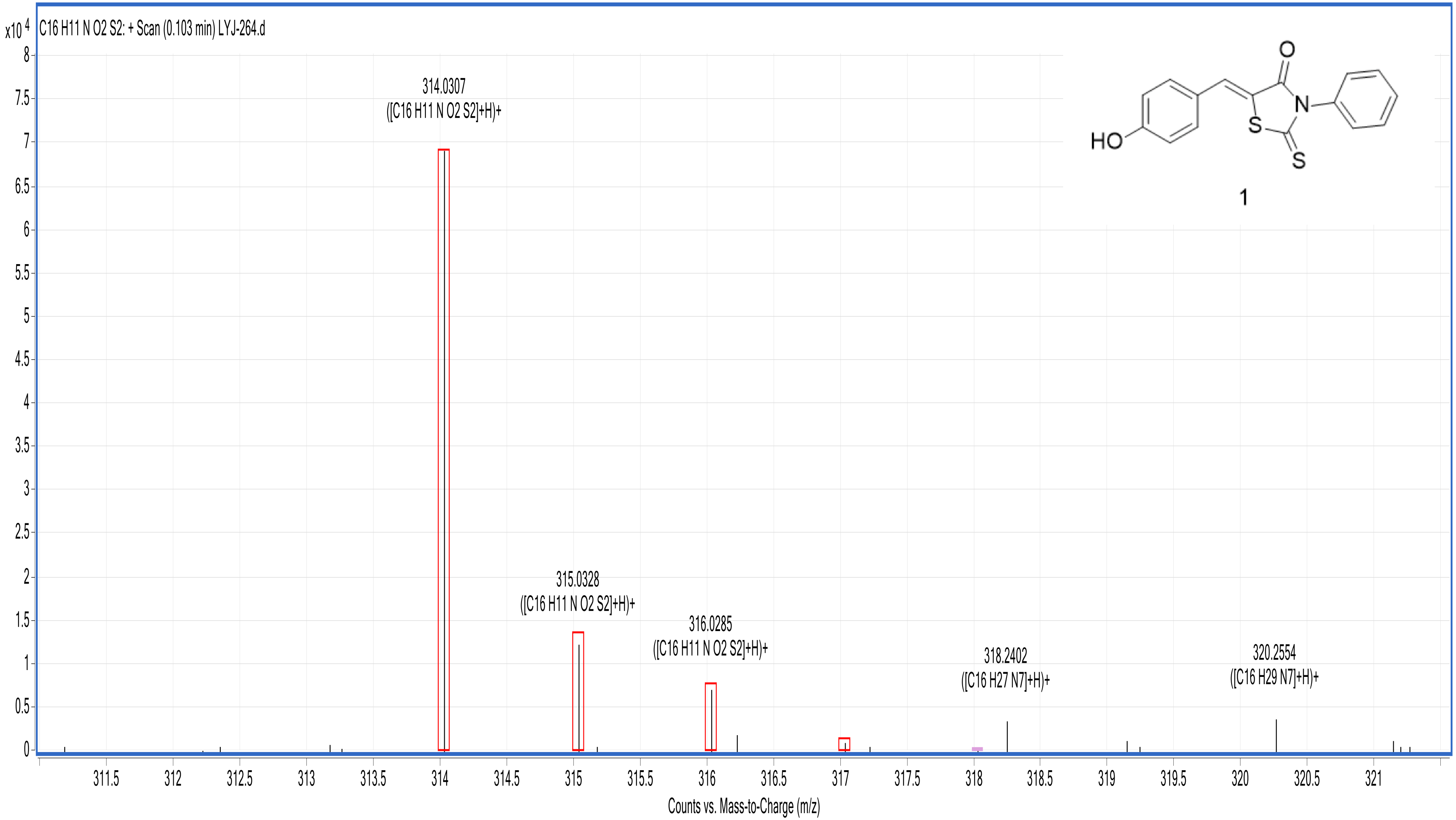

Figure S4. HRMS spectrum of compound **1**

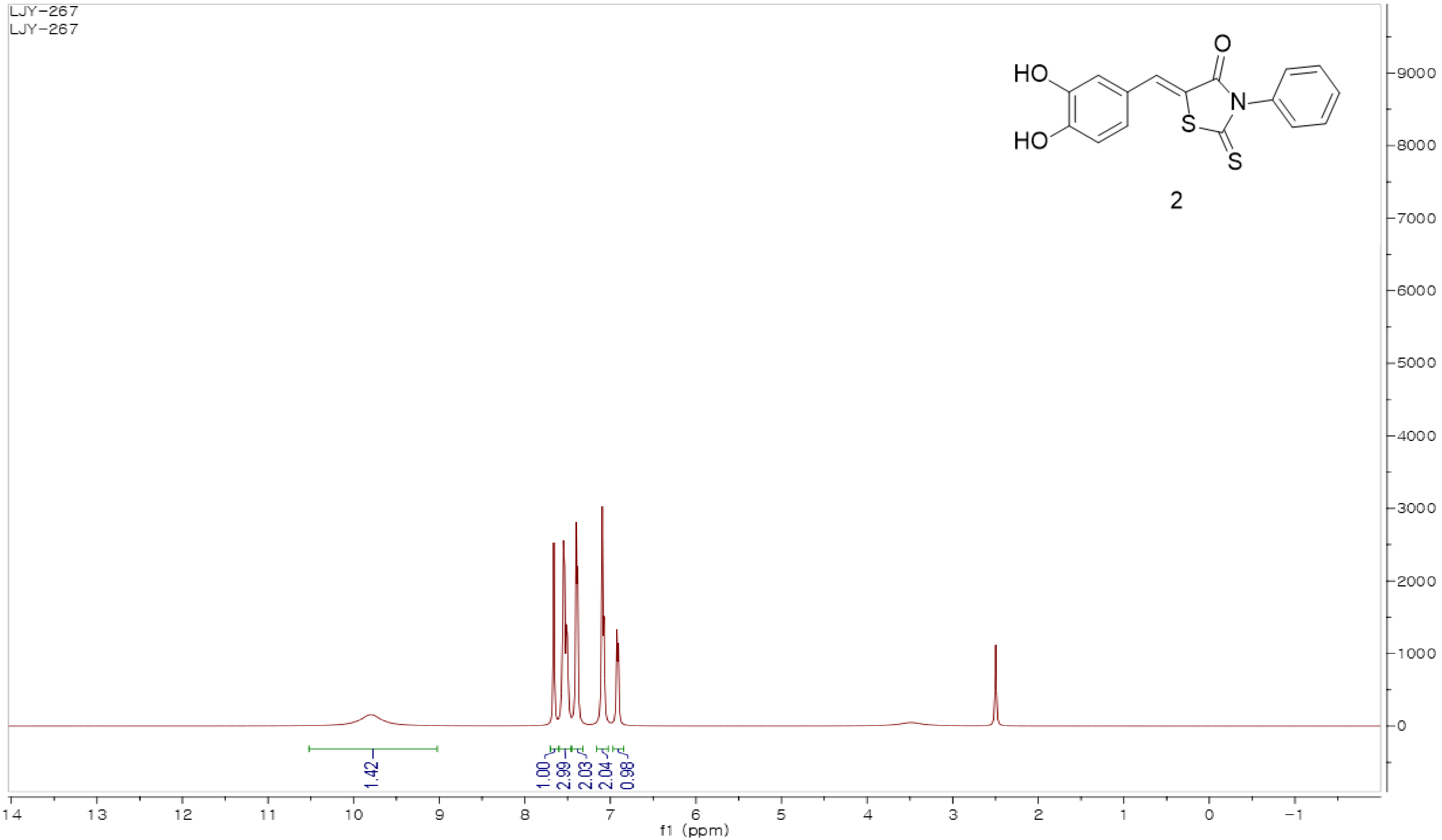

Figure S5. ^{1}H NMR spectrum of compound **2**

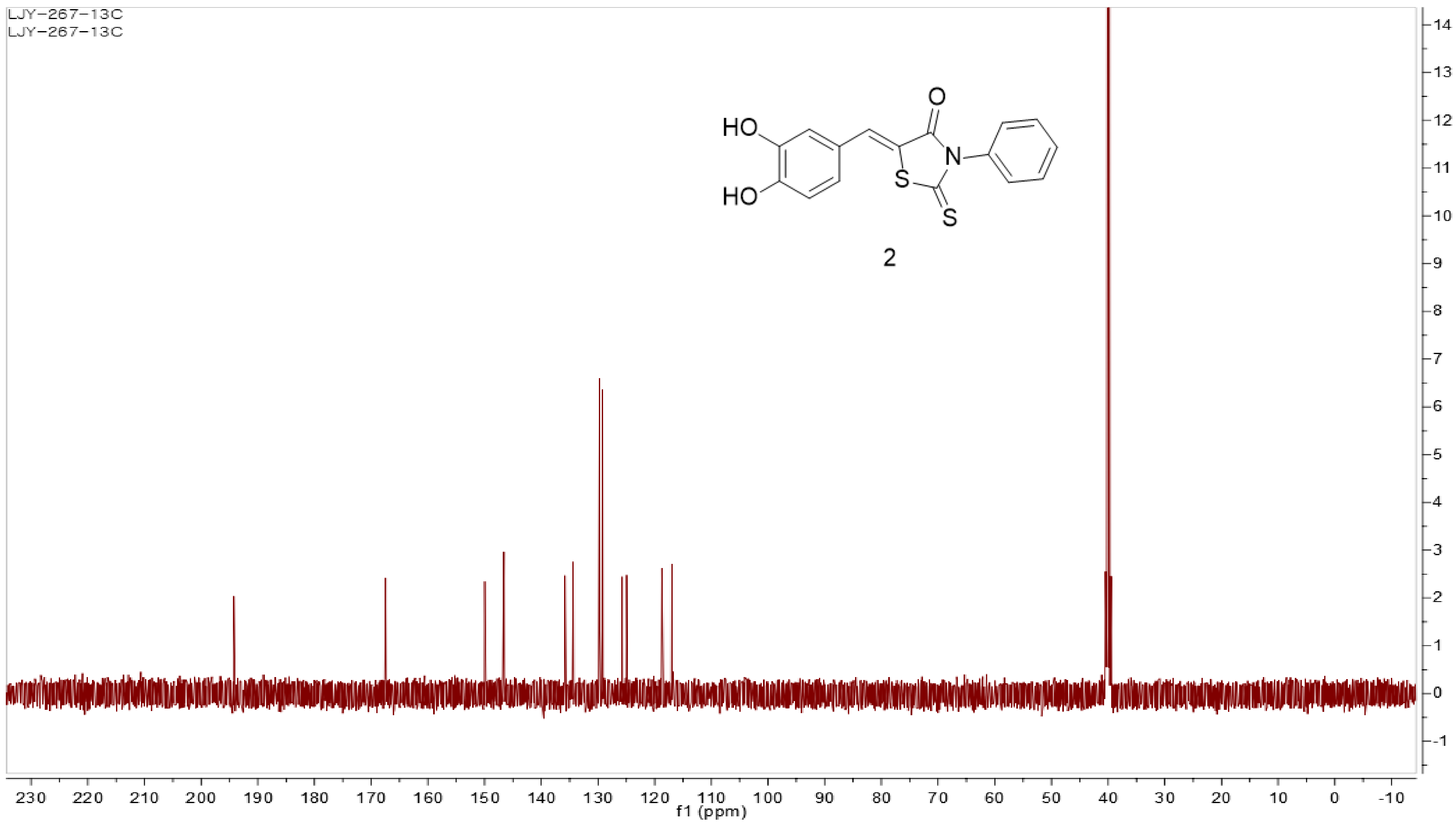

Figure S6. ^{13}C NMR spectrum of compound **2**

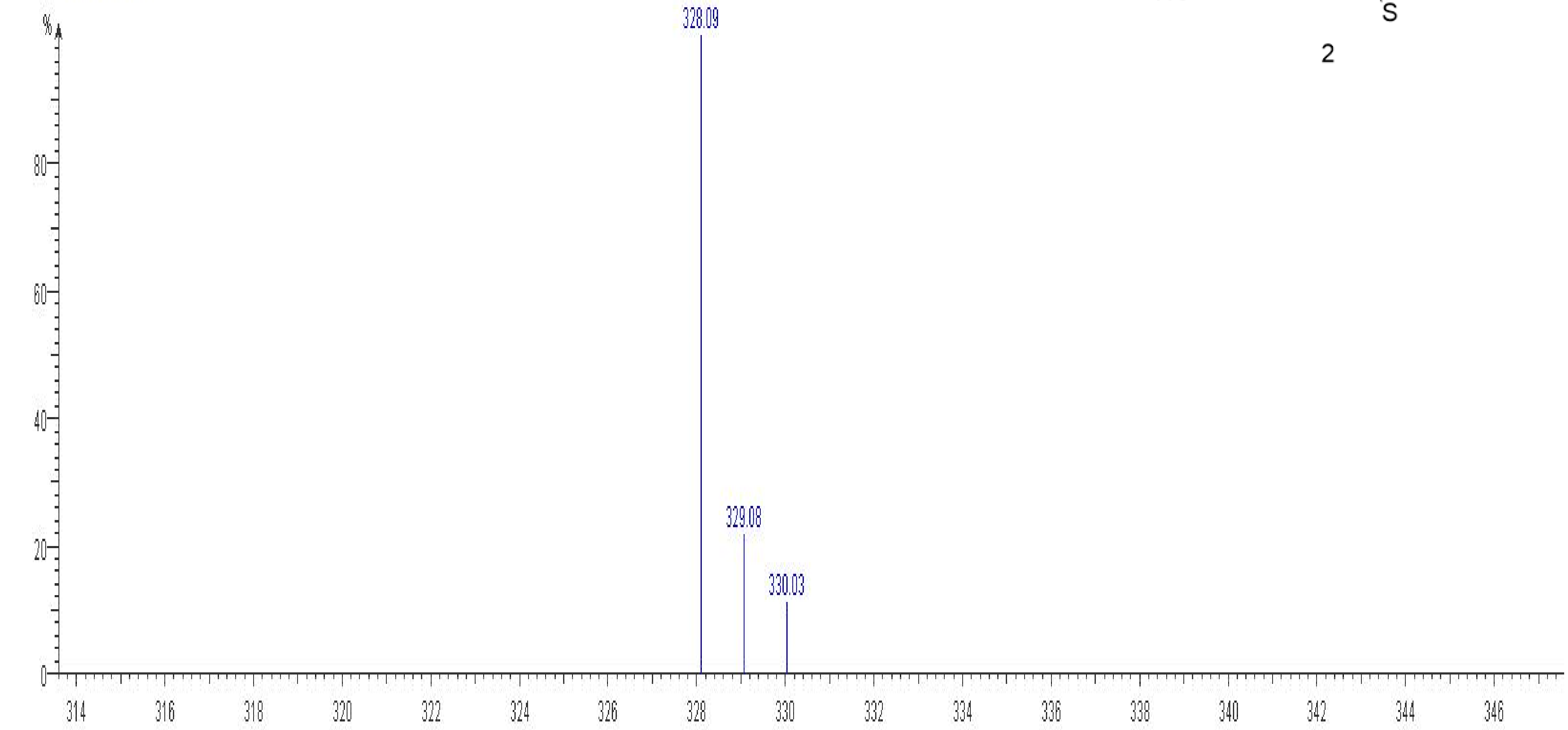

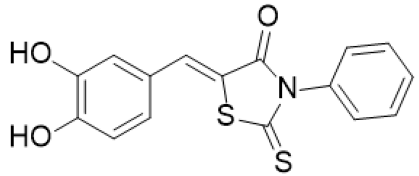

2

Figure S7. LRMS spectrum of compound **2**

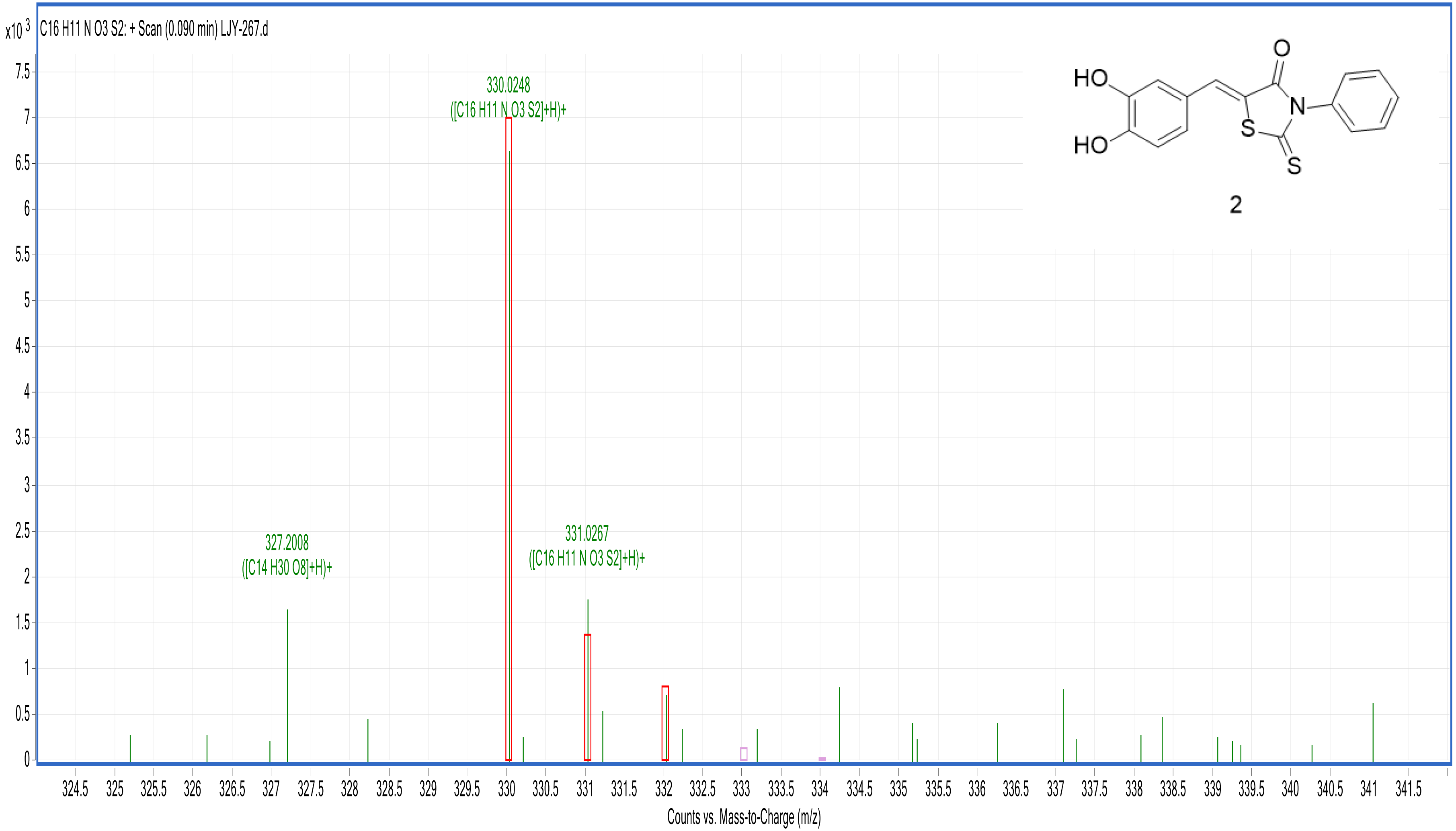

Figure S8. HRMS spectrum of compound **2**

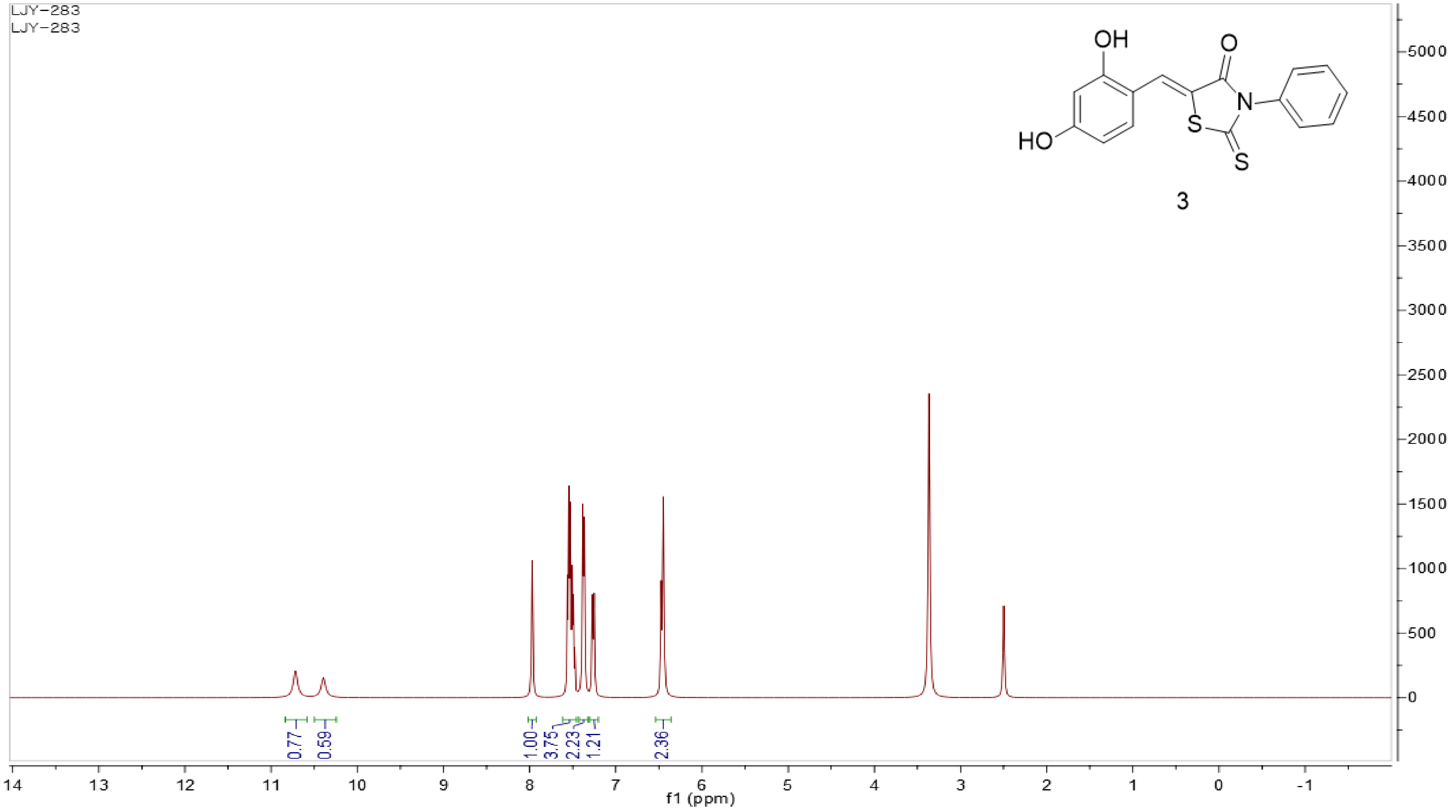

Figure S9. ^{1}H NMR spectrum of compound **3**

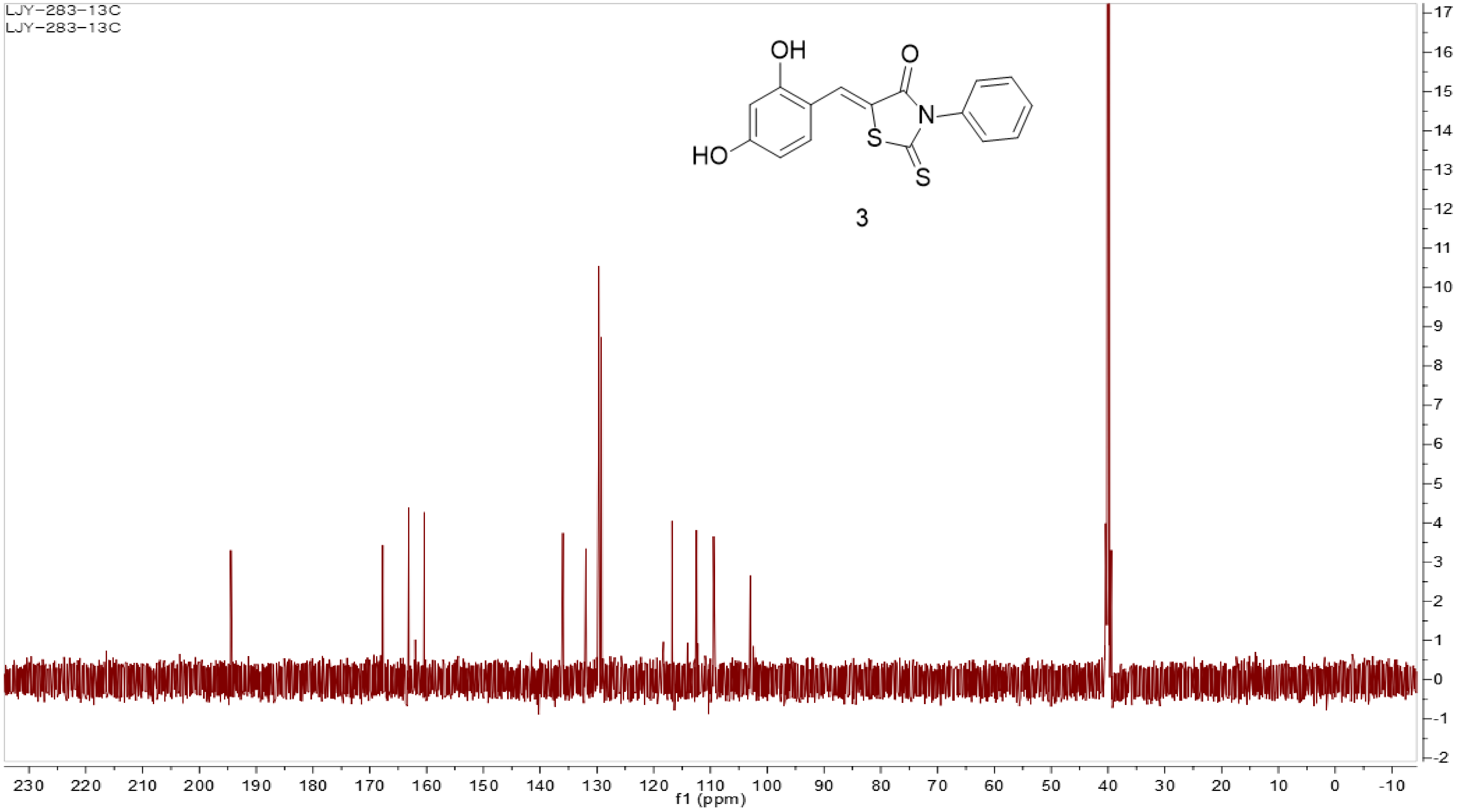

Figure S10. ^{13}C NMR spectrum of compound **3**

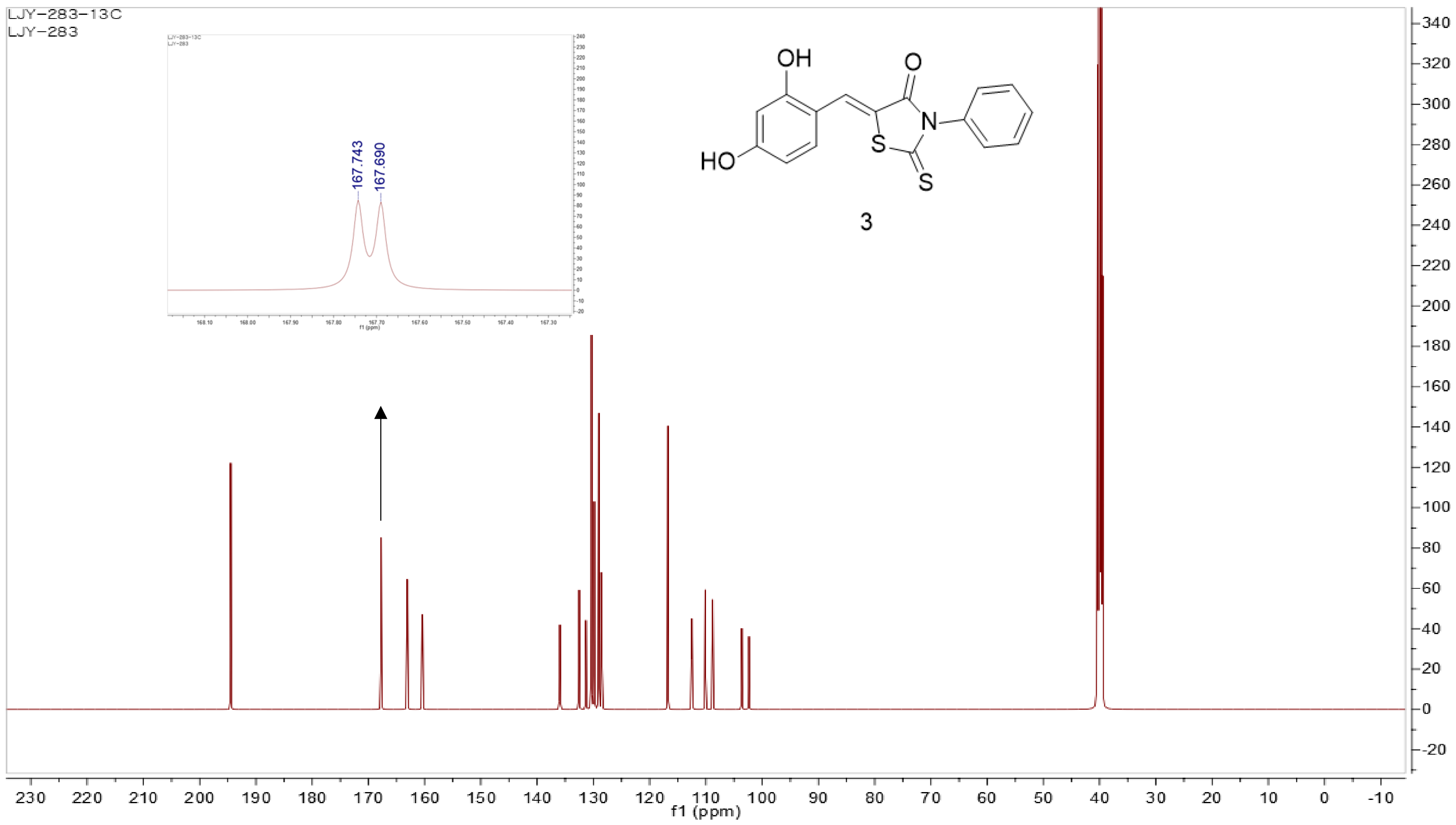

Figure S11. Proton-coupled ^{13}C NMR spectrum of compound **3**

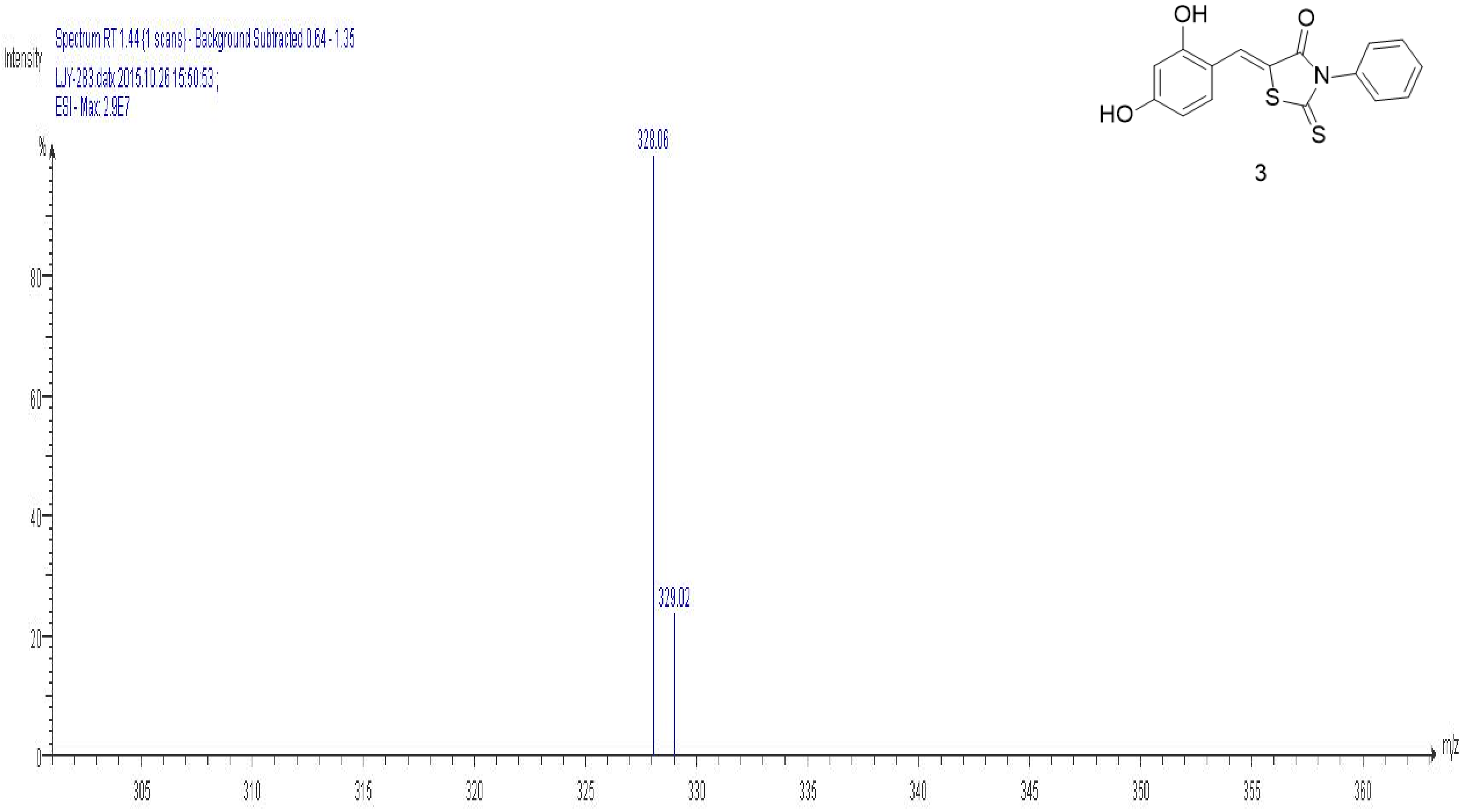

3

Figure S12. LRMS spectrum of compound **3**

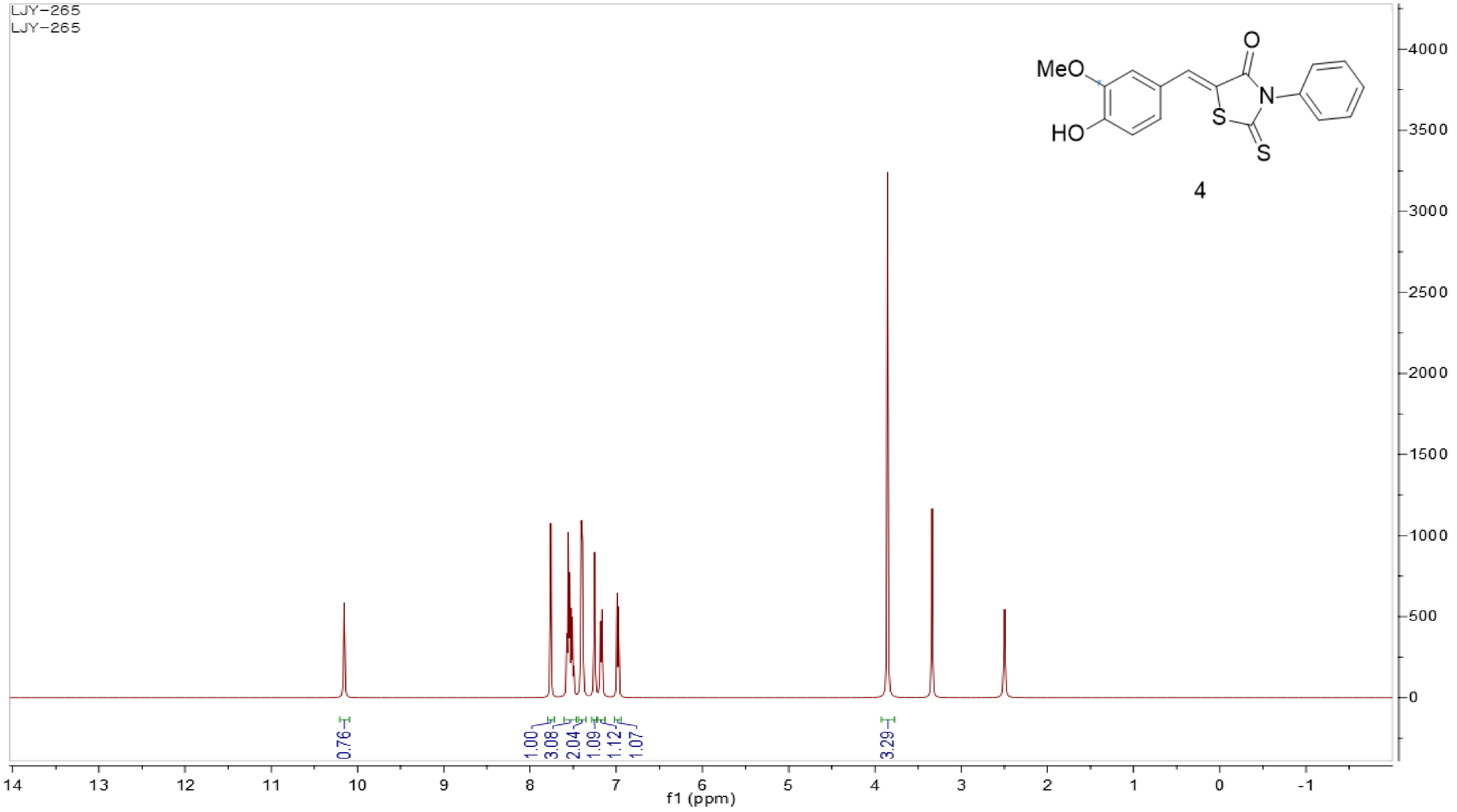

Figure S13. ^{1}H NMR spectrum of compound **4**

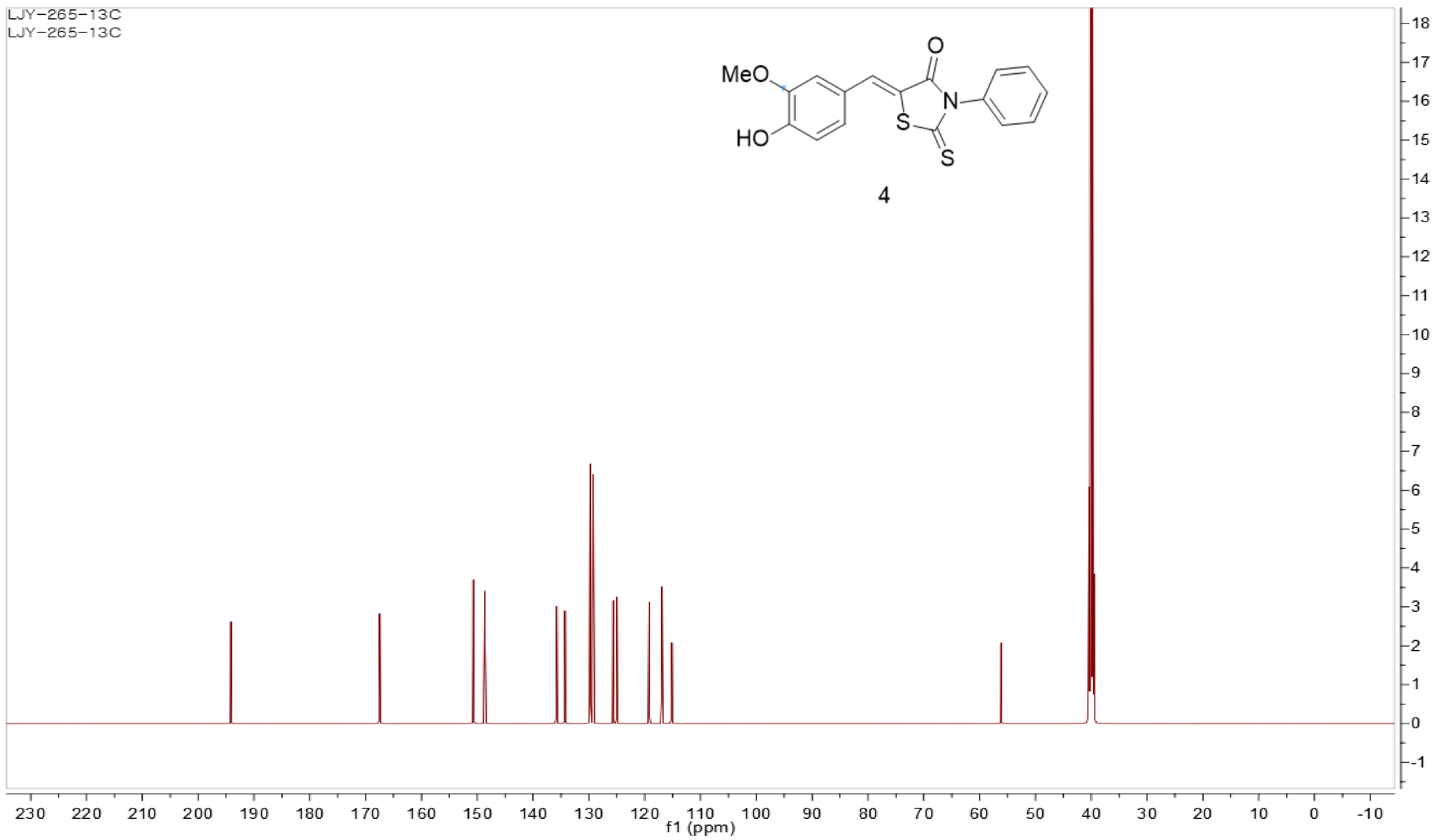

Figure S14. ^{13}C NMR spectrum of compound **4**

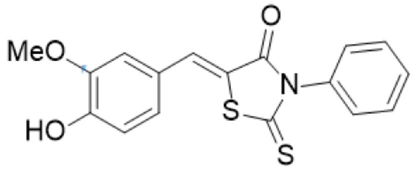

4

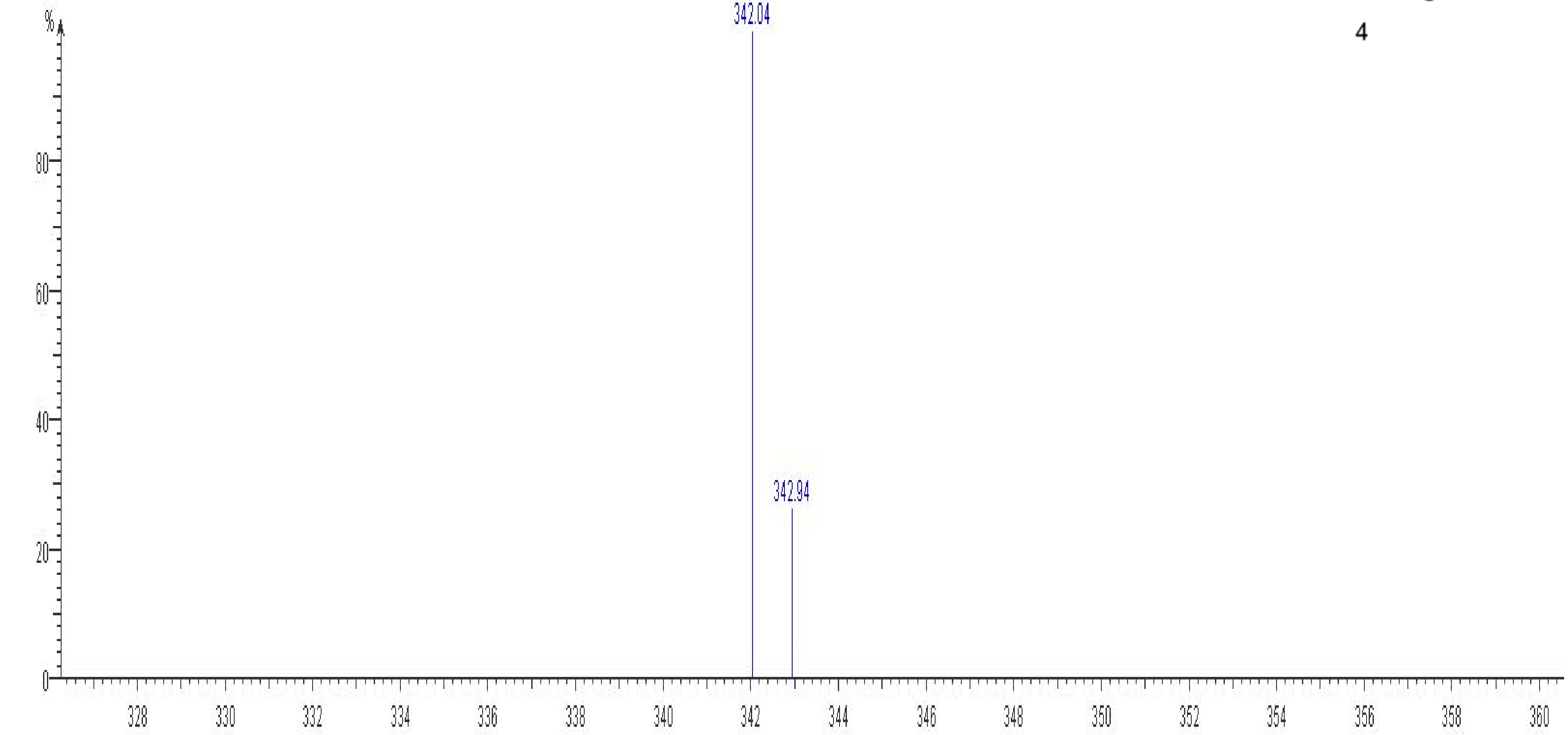

Figure S15. LRMS spectrum of compound **4**

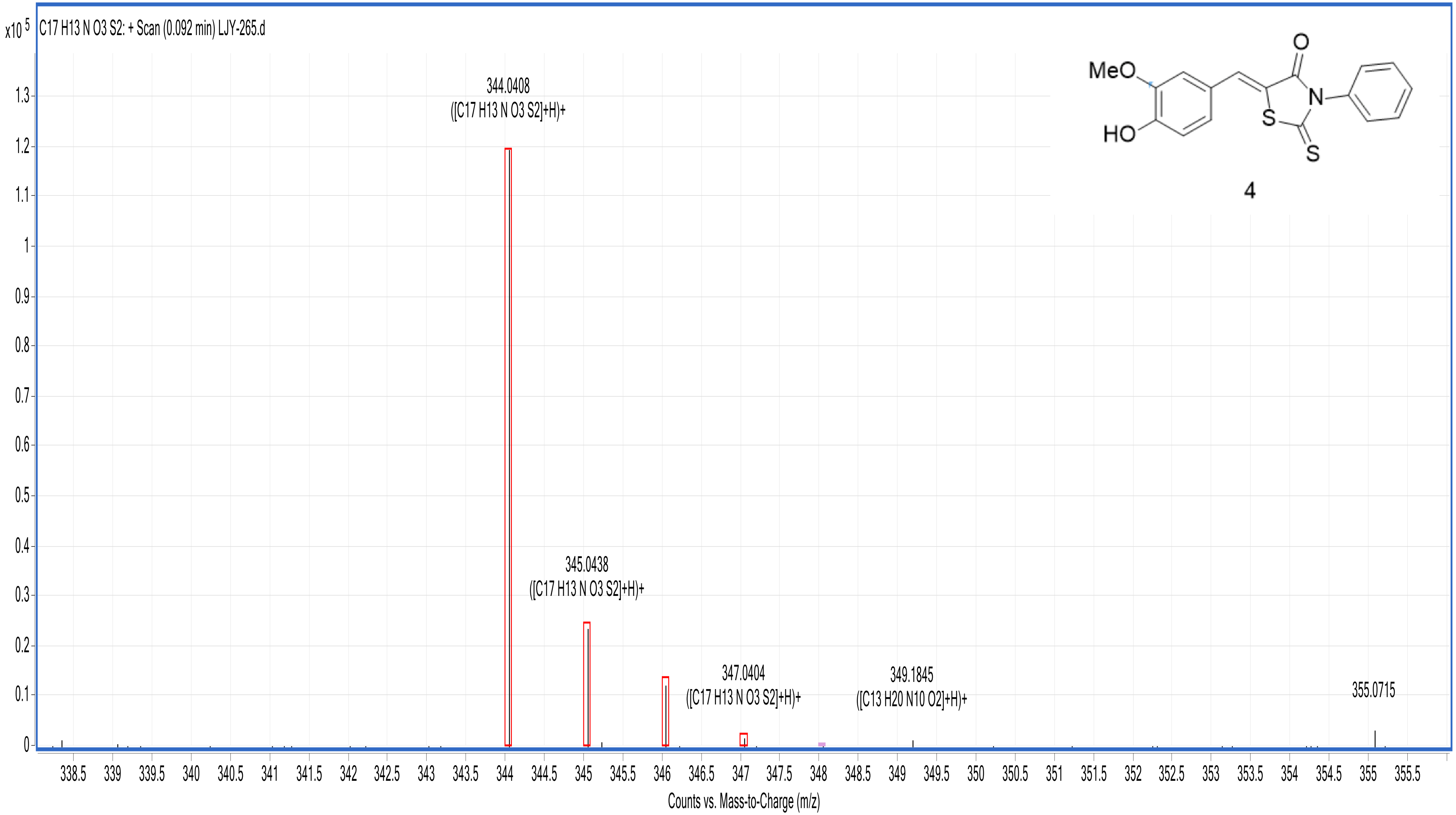

Figure S16. HRMS spectrum of compound **4**

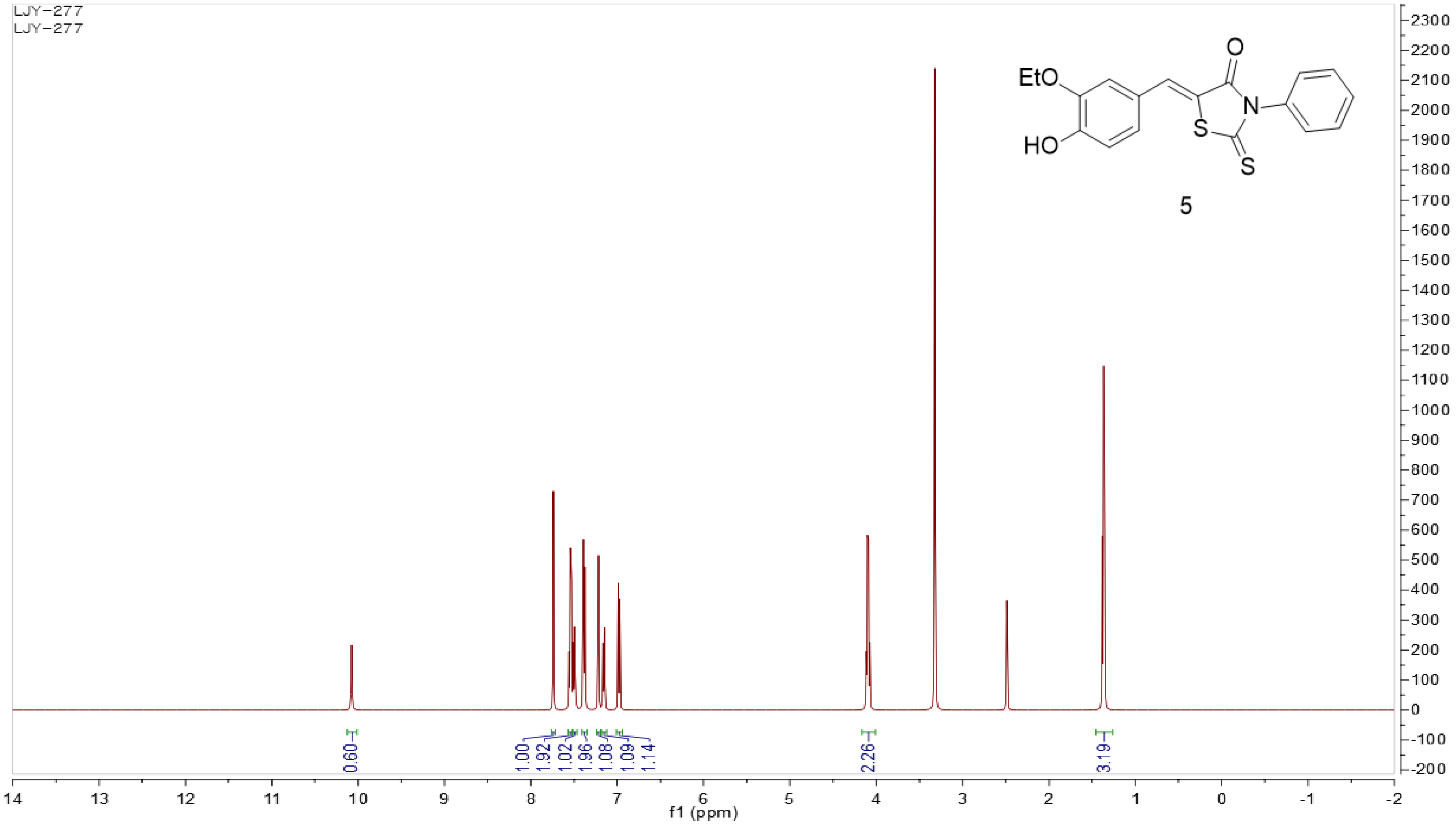

Figure S17. ^{1}H NMR spectrum of compound **5**

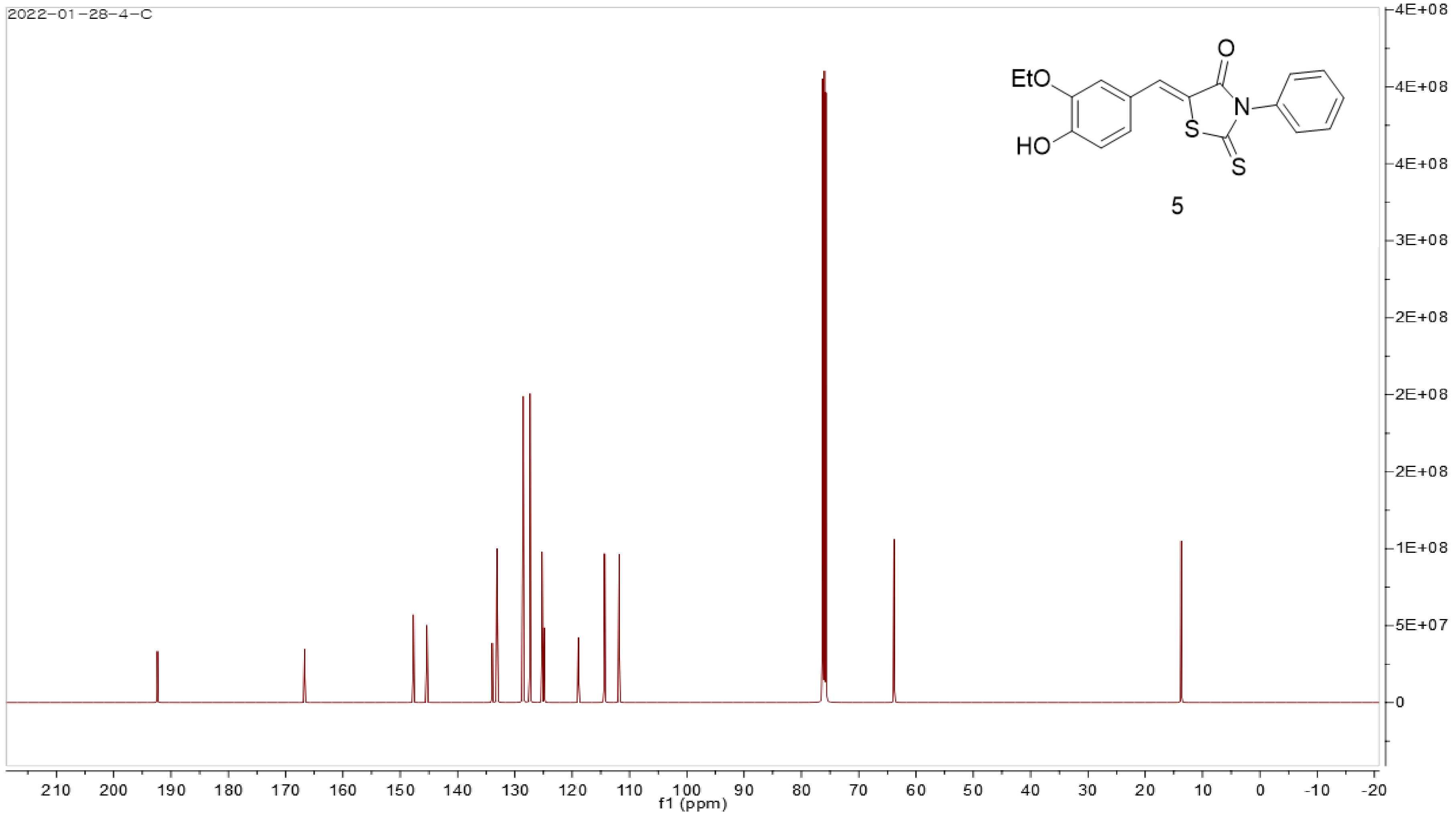

Figure S18. ^{13}C NMR spectrum of compound **5**

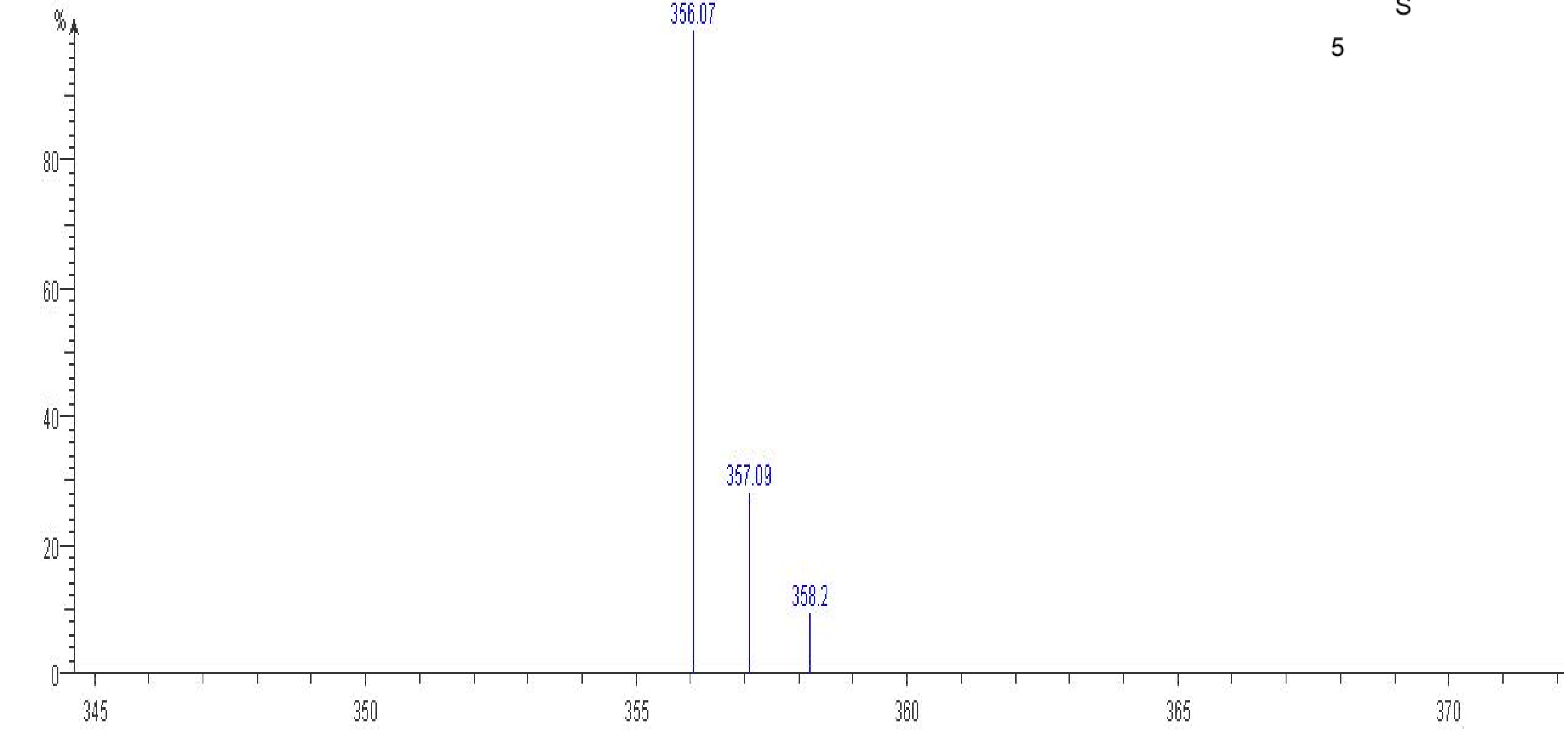

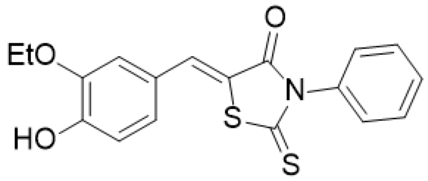

5

Figure S19. LRMS spectrum of compound **5**

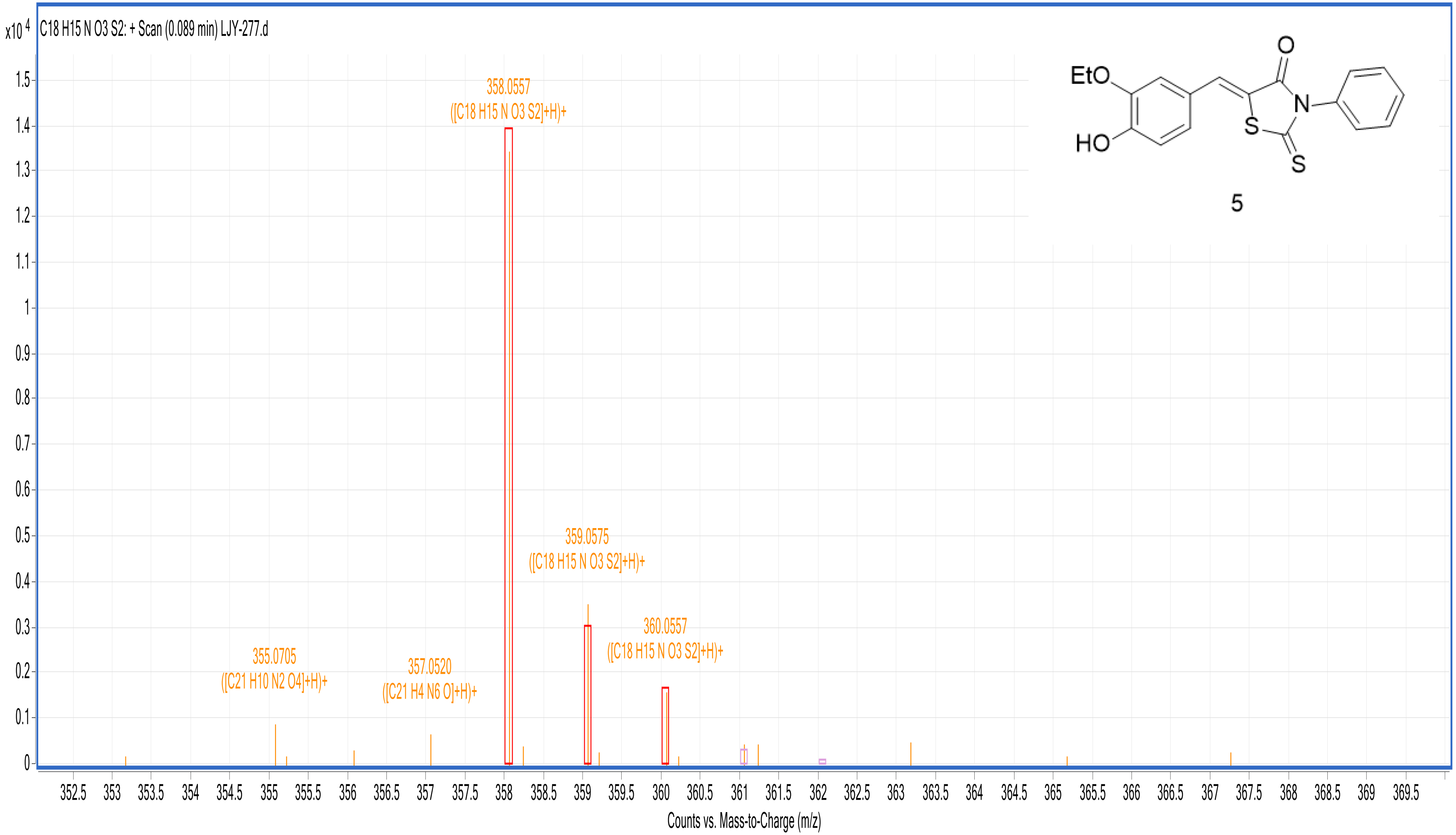

Figure S20. HRMS spectrum of compound **5**

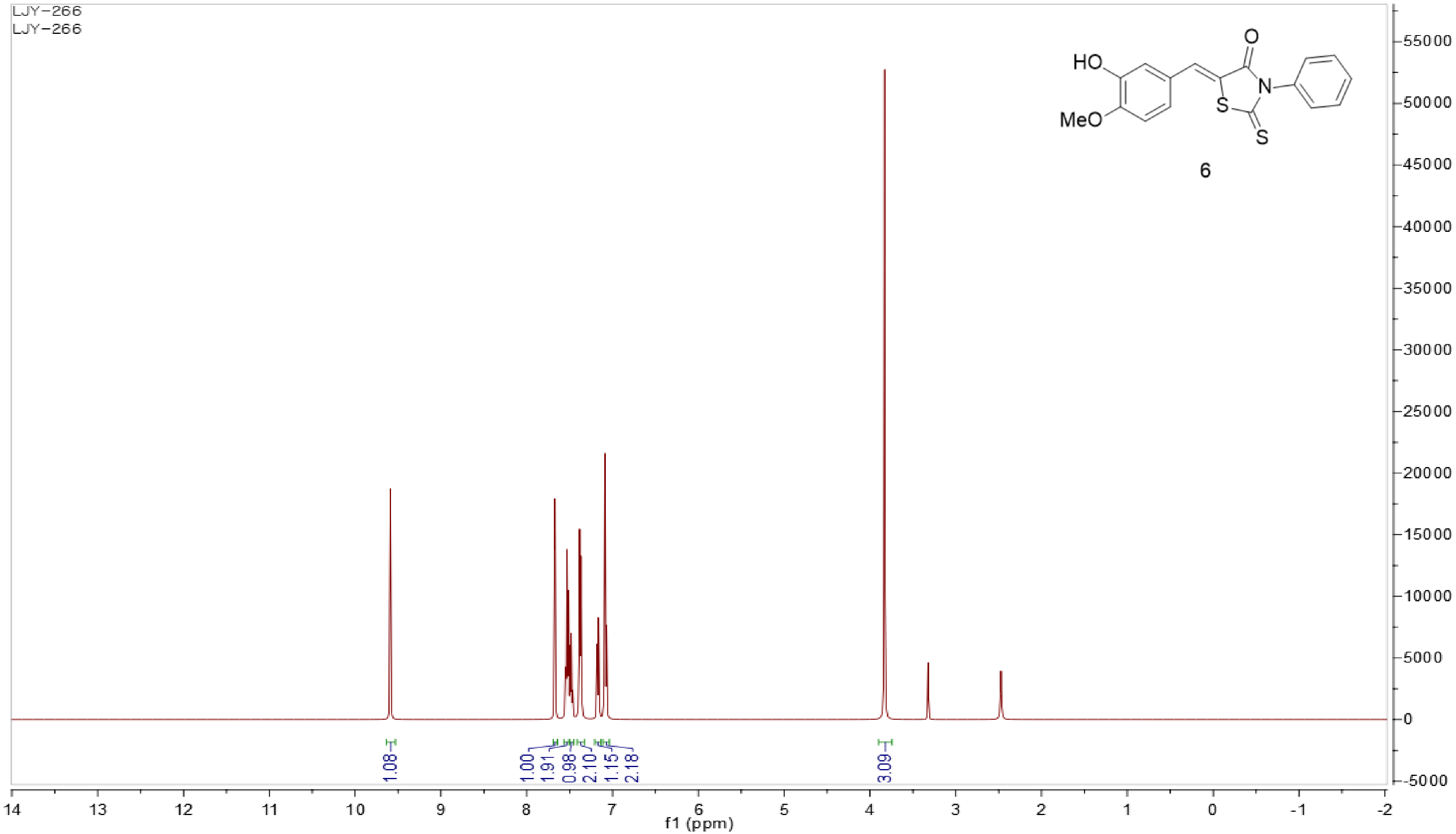

Figure S21. ^{1}H NMR spectrum of compound **6**

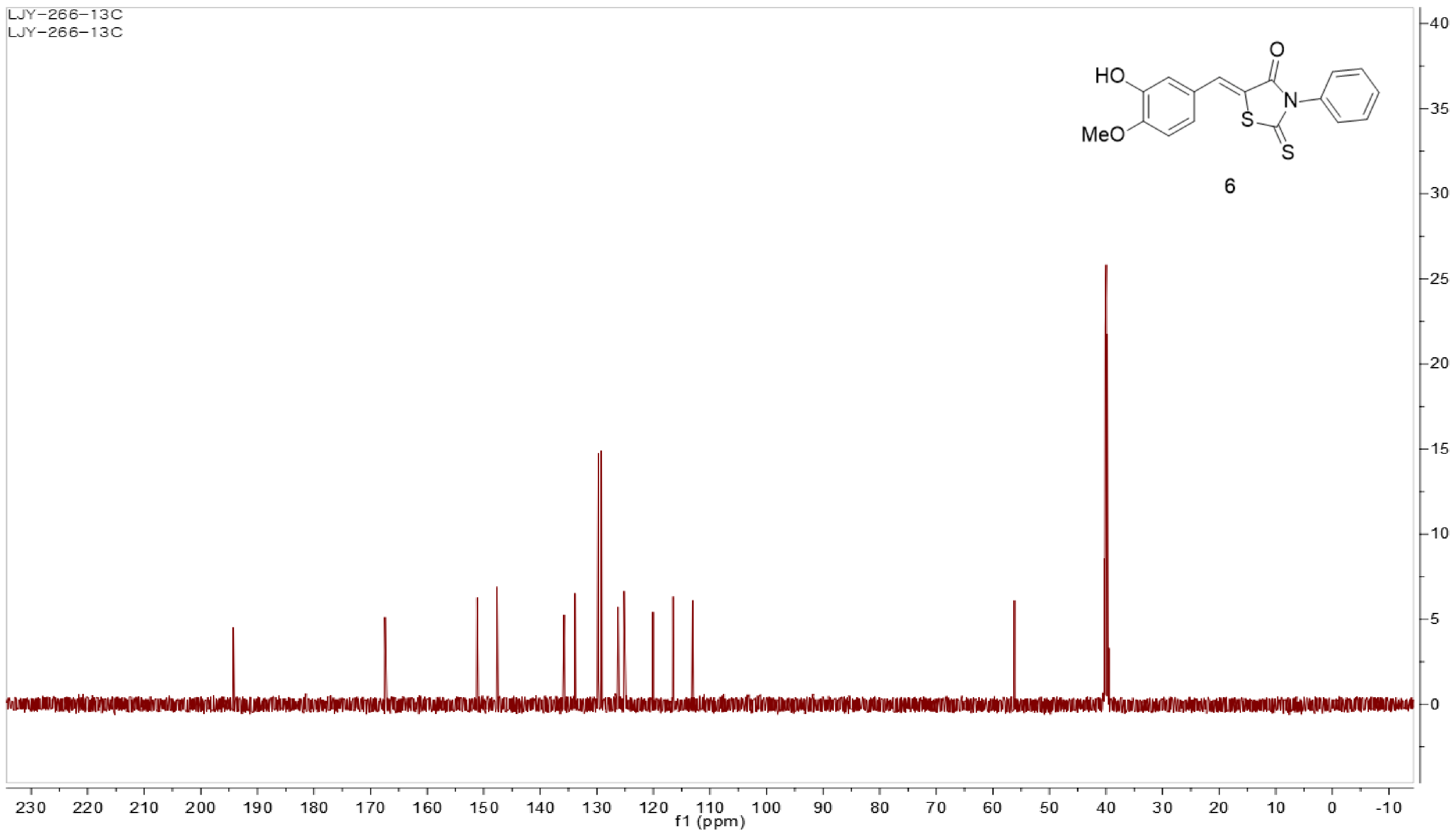

Figure S22. ^{13}C NMR spectrum of compound **6**

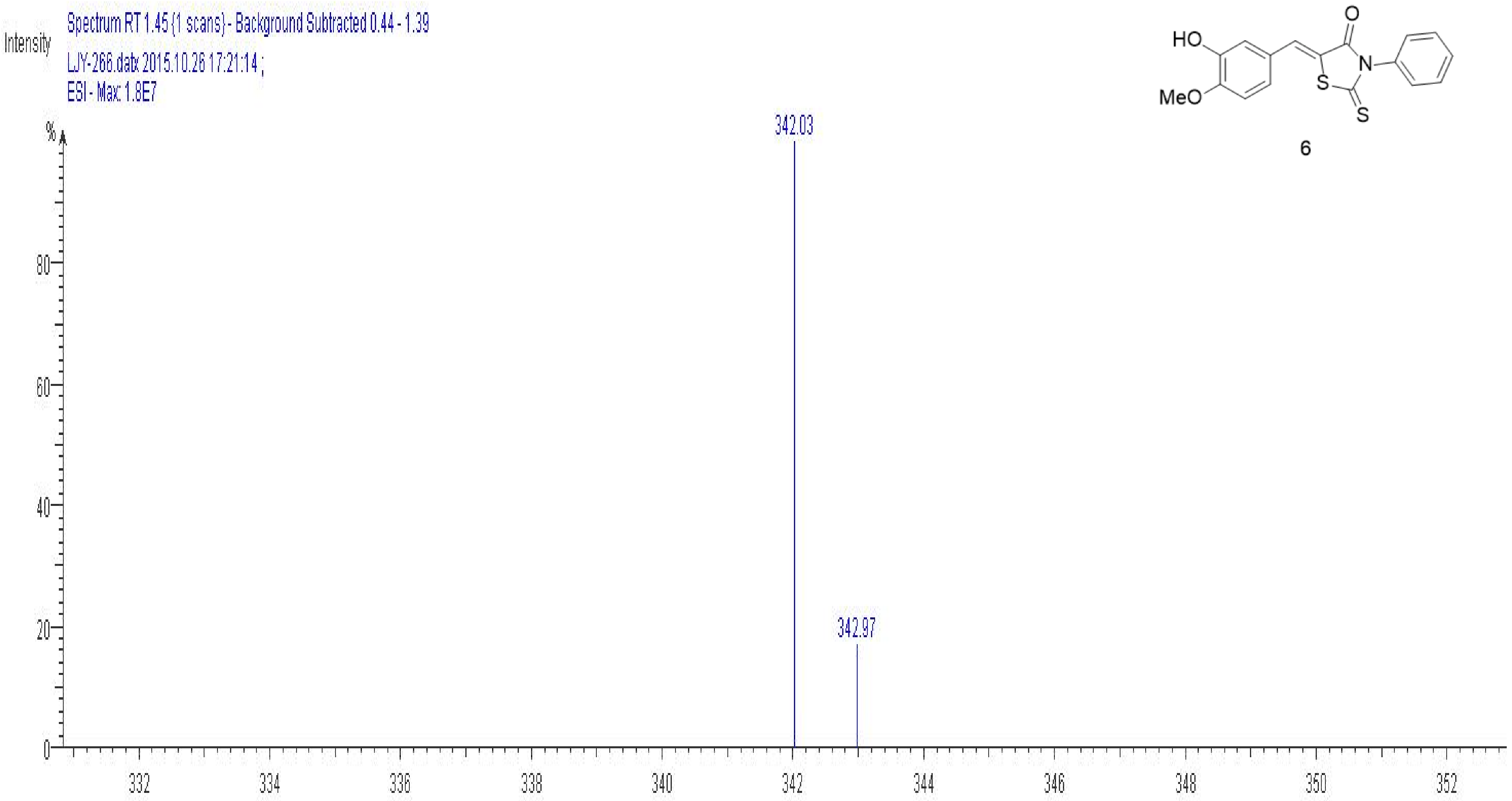

Figure S23. LRMS spectrum of compound **6**

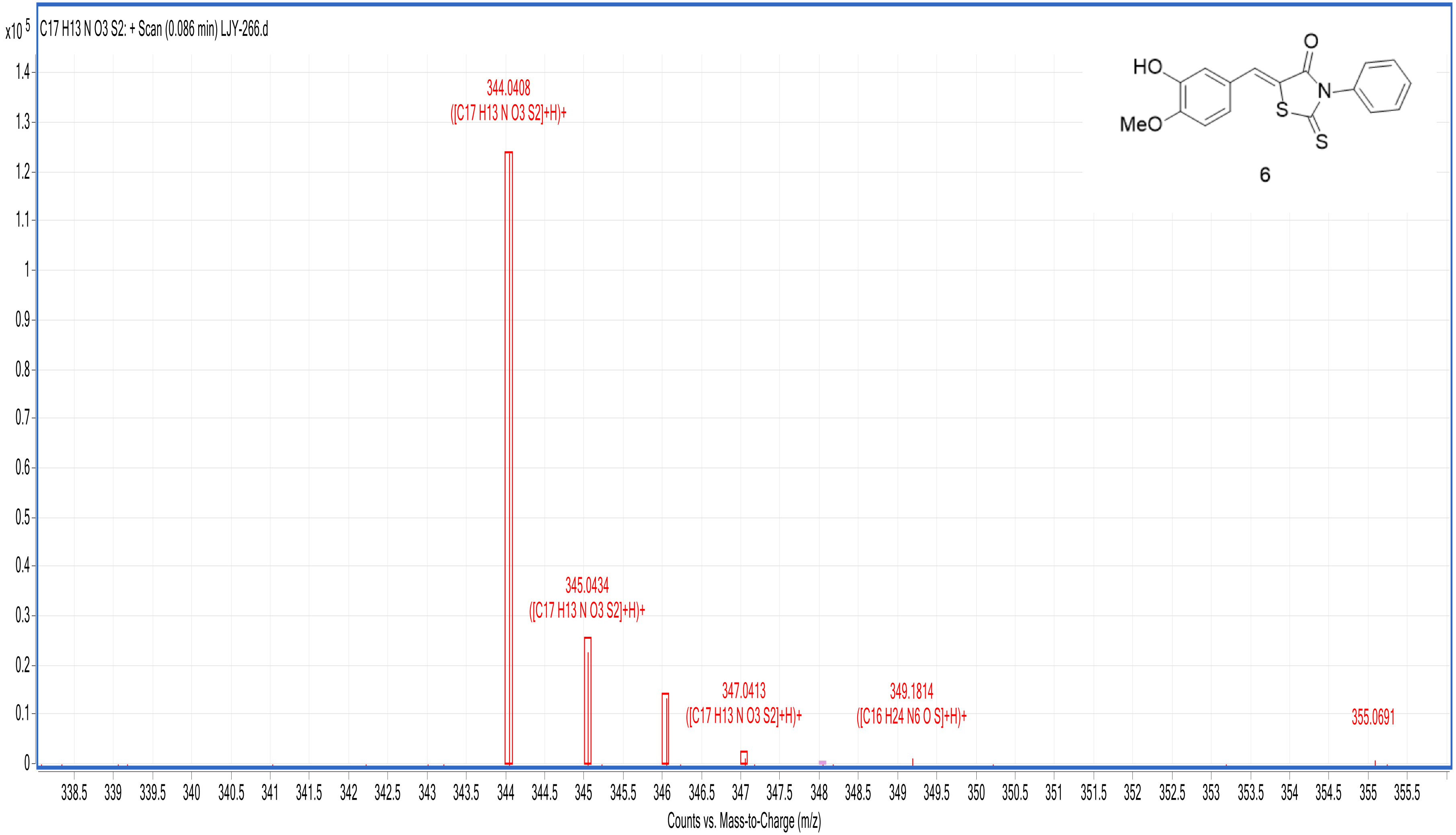

Figure S24. HRMS spectrum of compound **6**

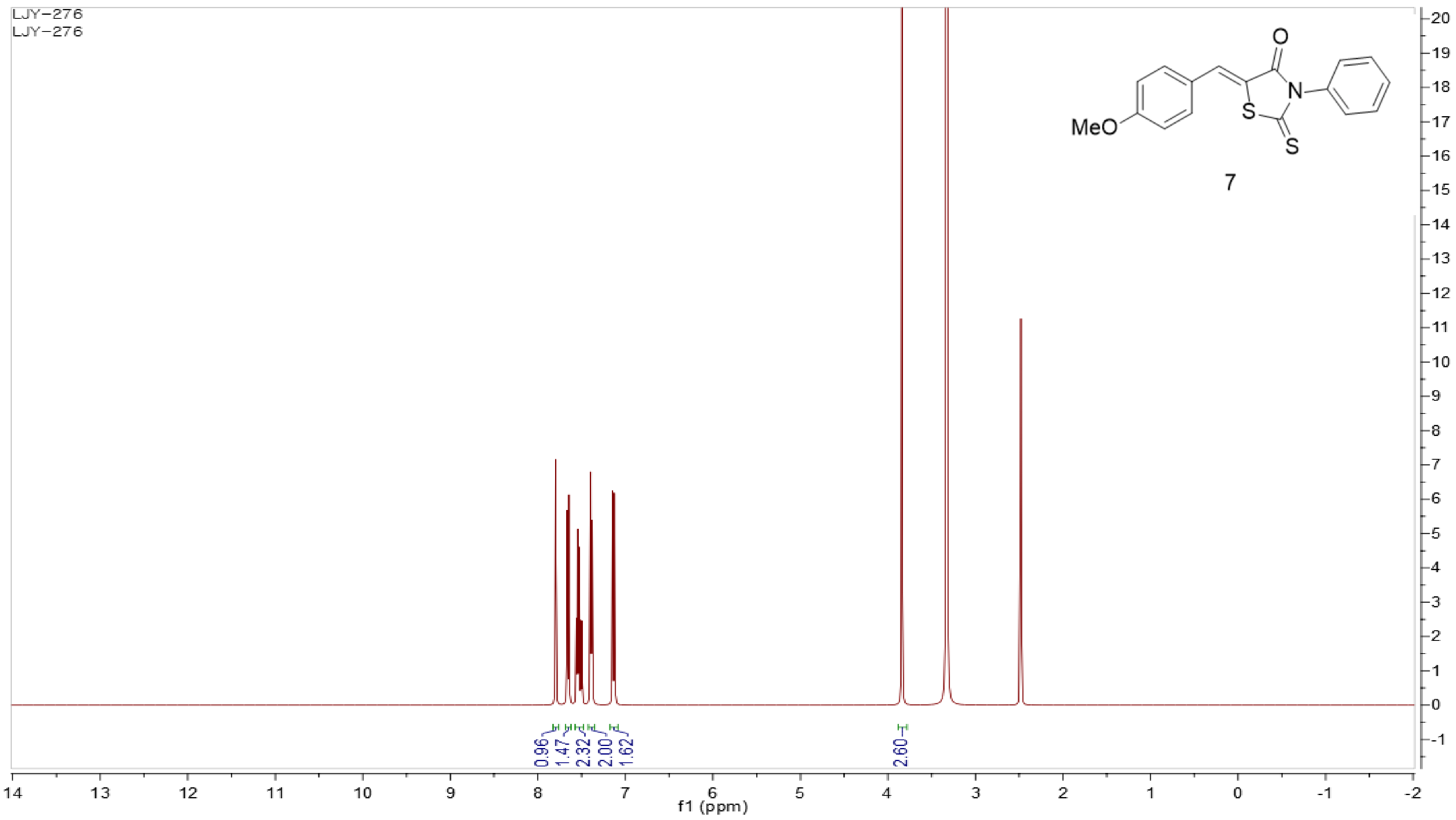

Figure S25. ^{1}H NMR spectrum of compound **7**

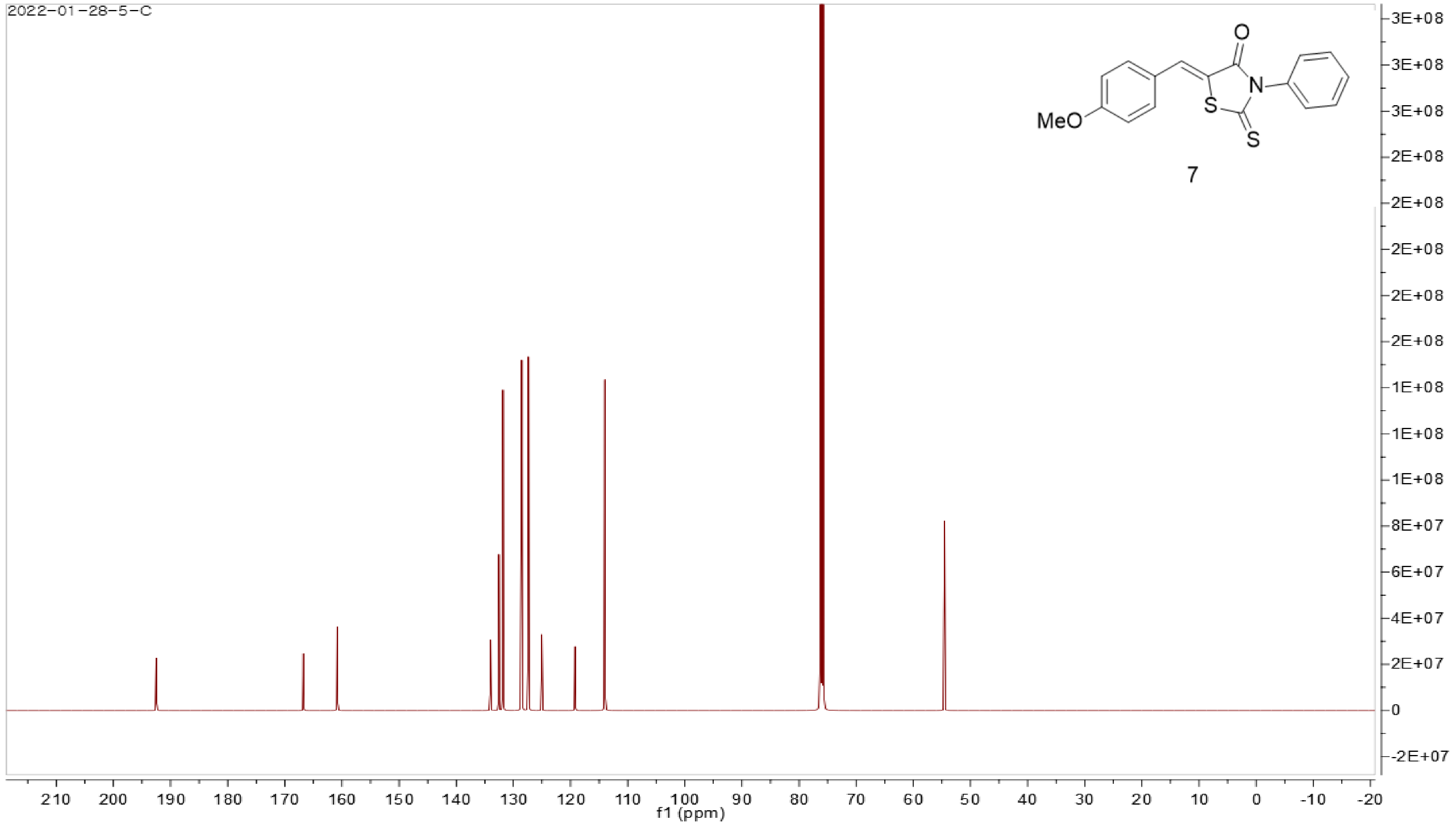

Figure S26. ^{13}C NMR spectrum of compound **7**

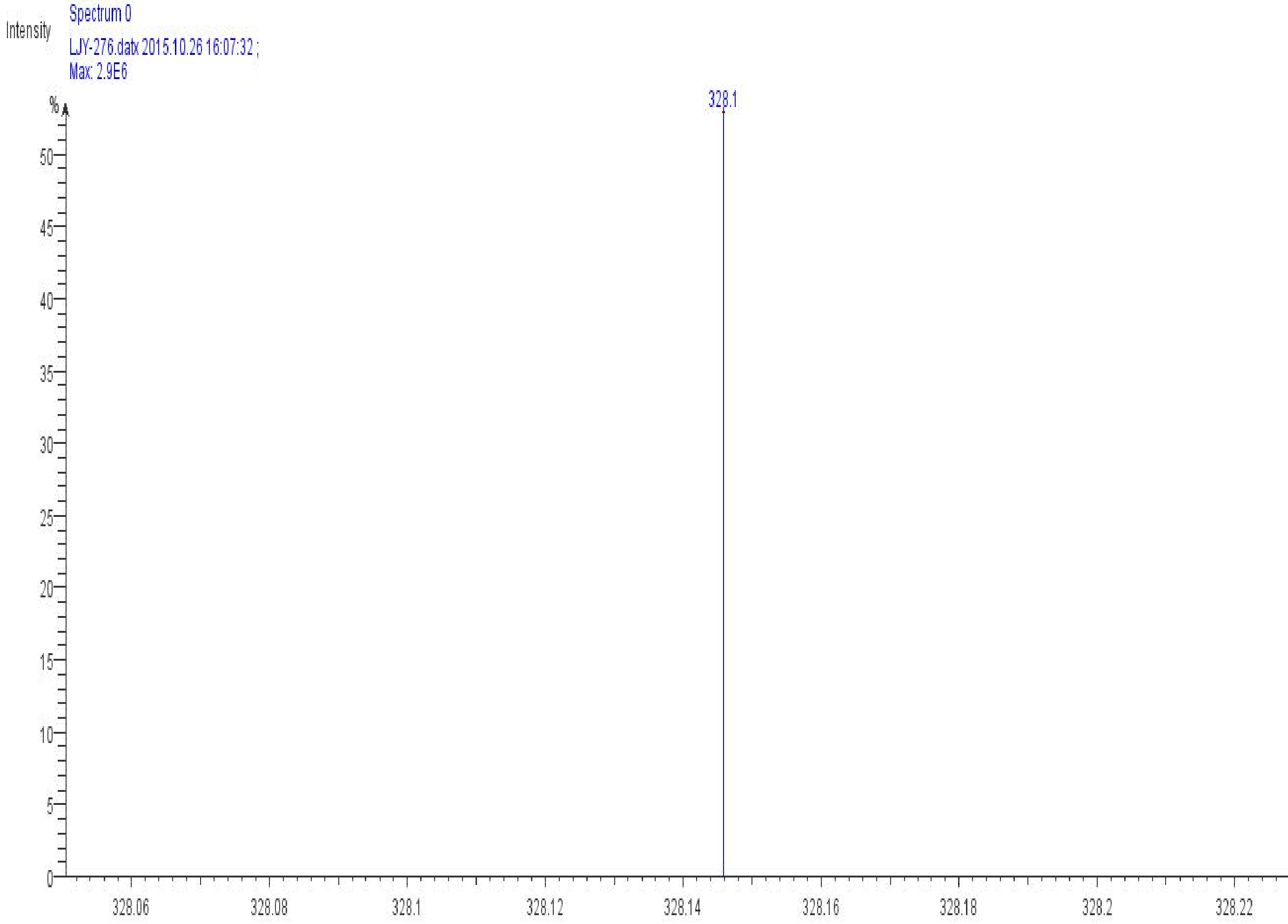

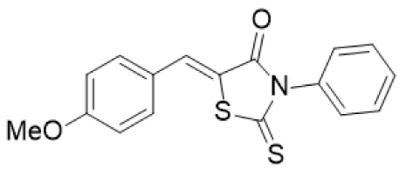

7

Figure S27. LRMS spectrum of compound **7**

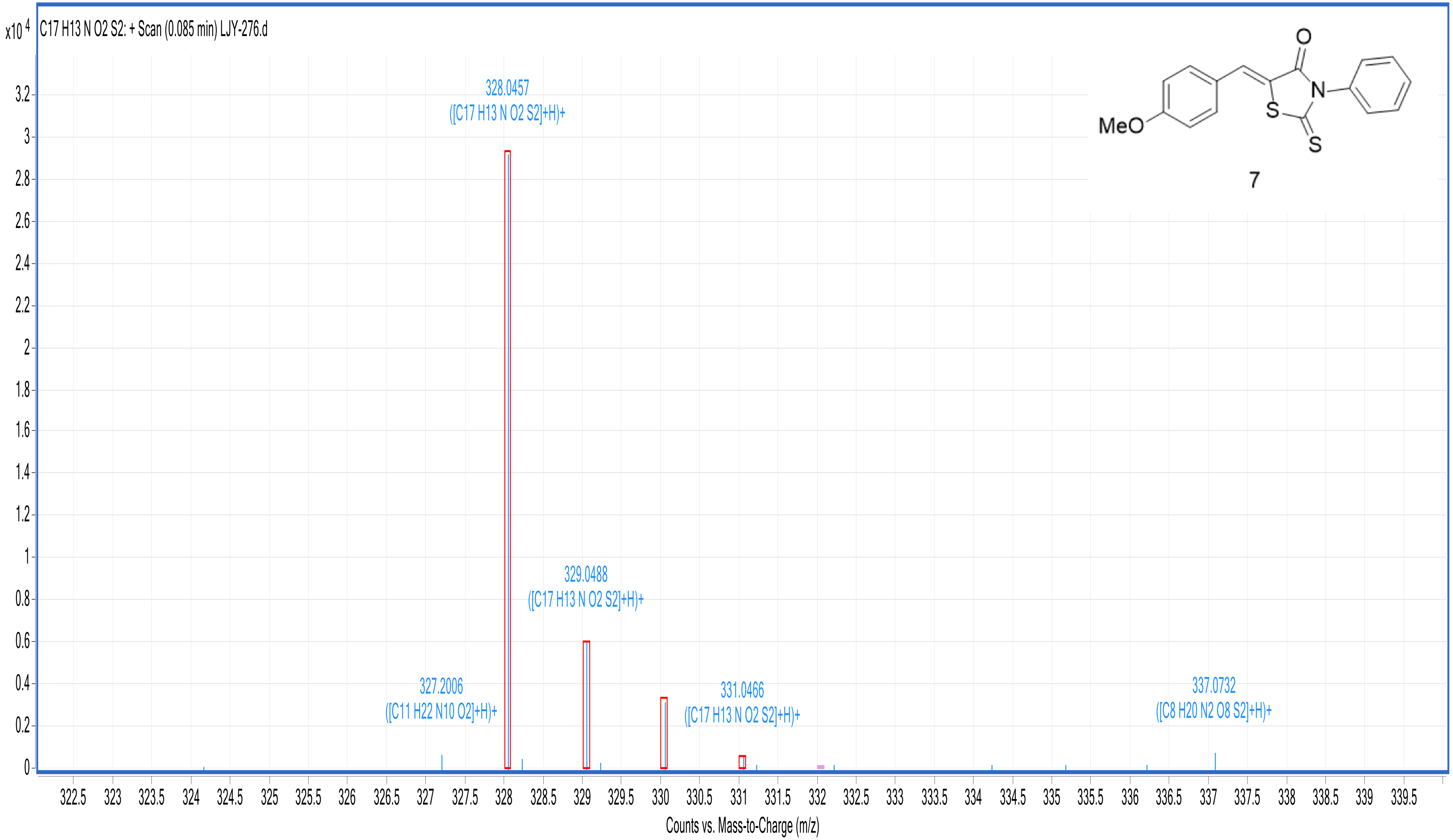

Figure S28. HRMS spectrum of compound **7**

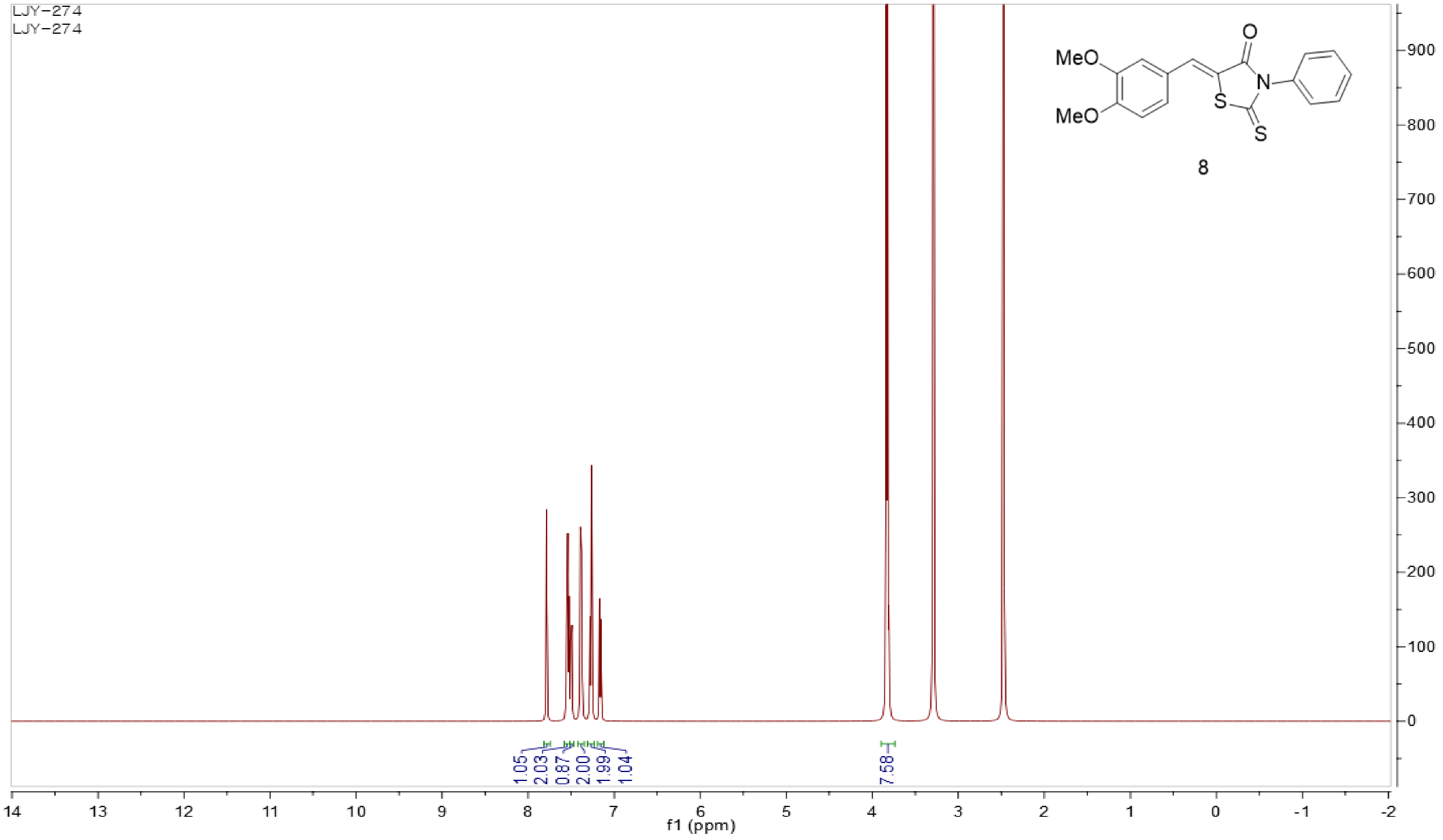

Figure S29. ^{1}H NMR spectrum of compound **8**

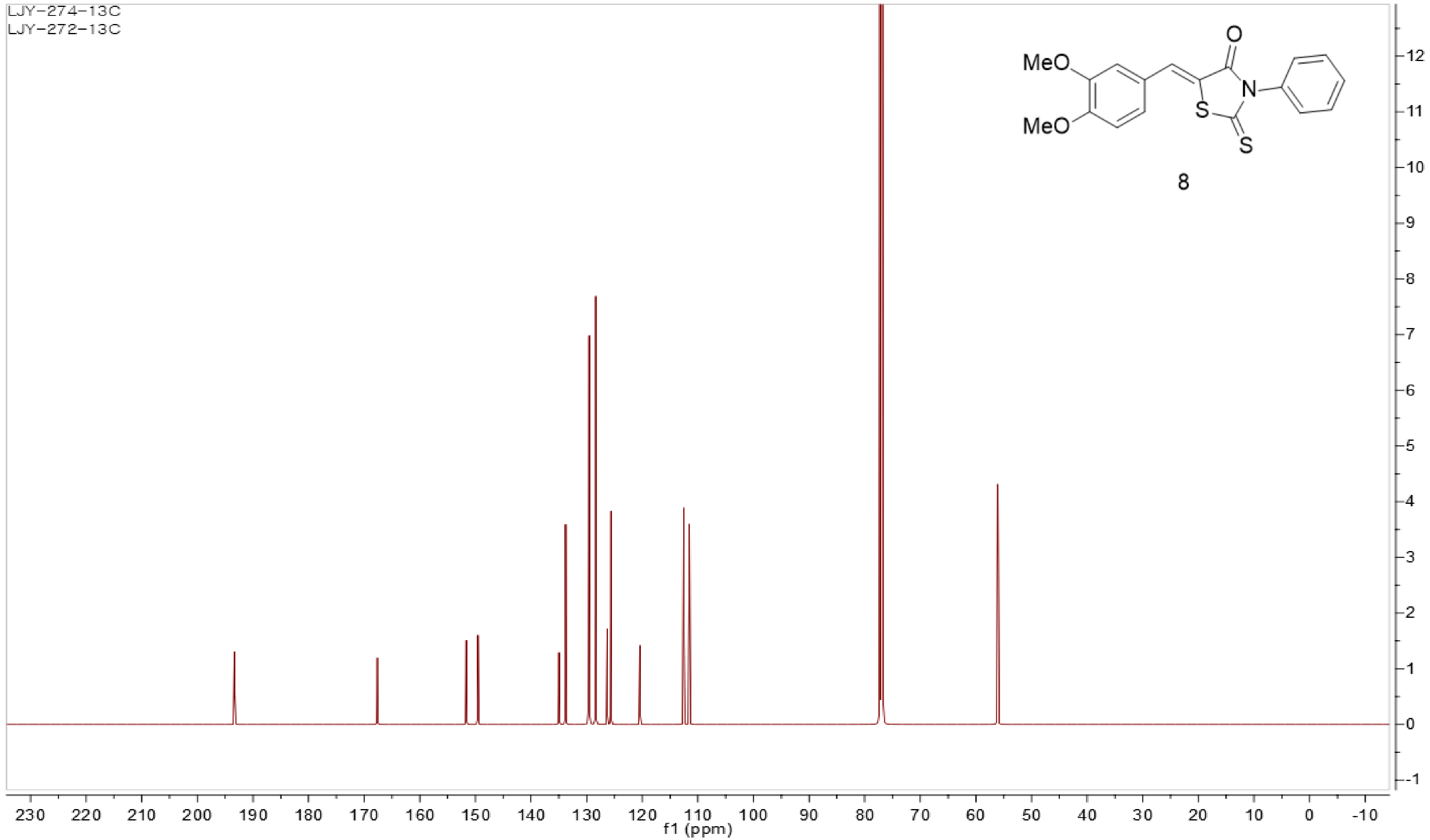

Figure S30. ^{13}C NMR spectrum of compound **8**

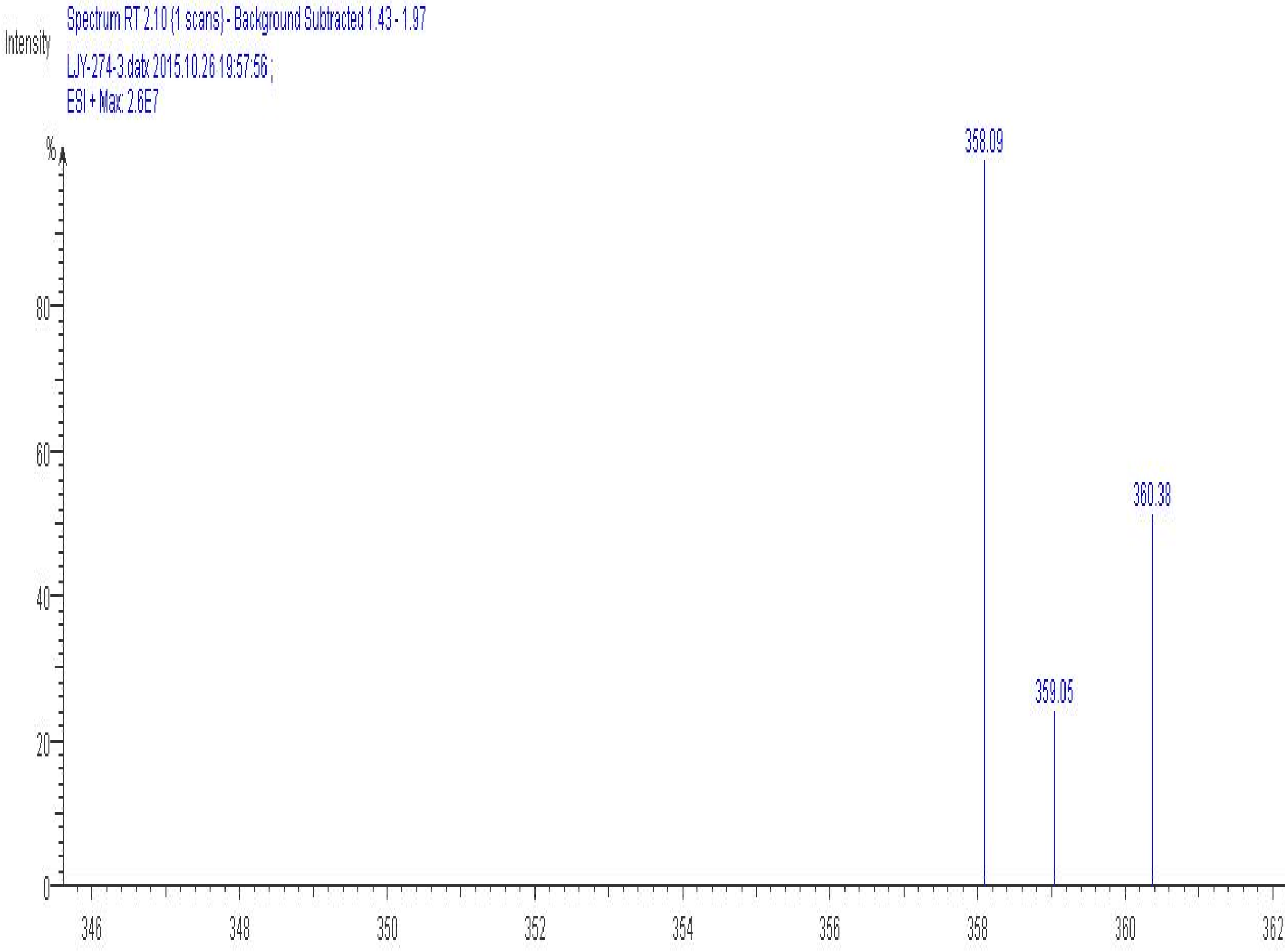

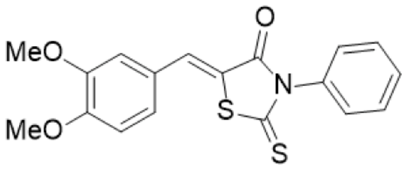

8

Figure S31. LRMS spectrum of compound **8**

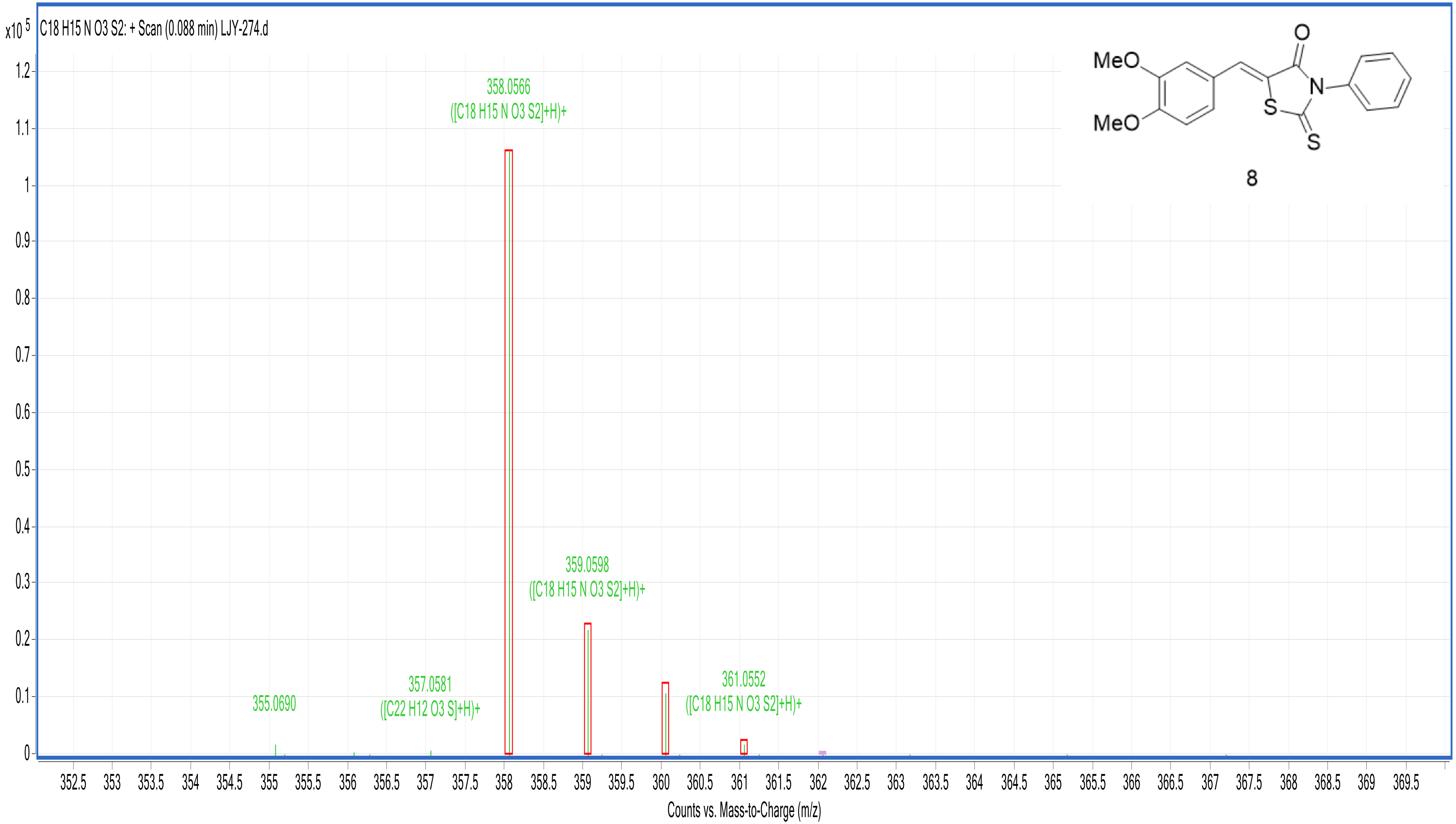

Figure S32. HRMS spectrum of compound **8**

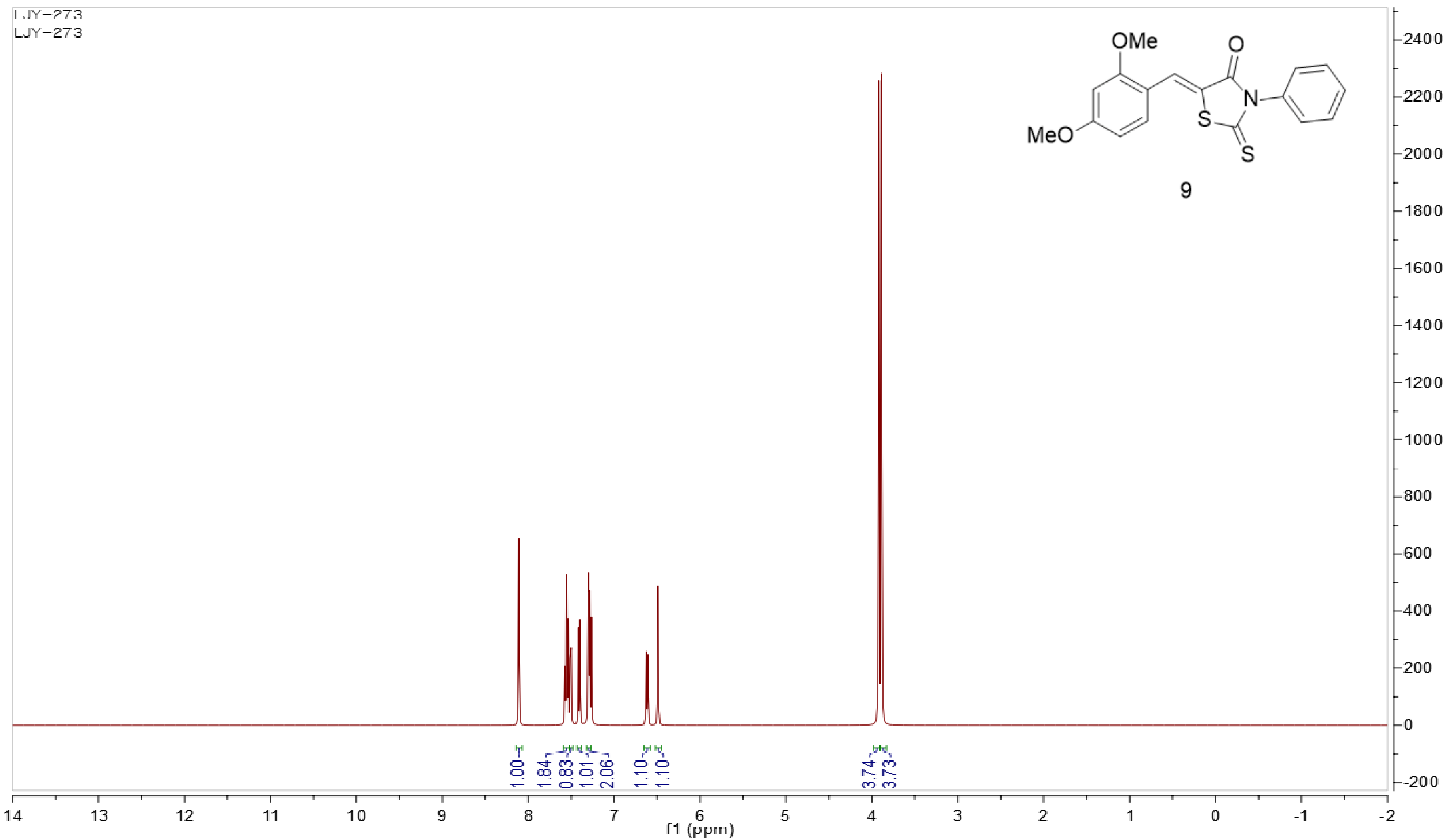

Figure S33. ^{1}H NMR spectrum of compound **9**

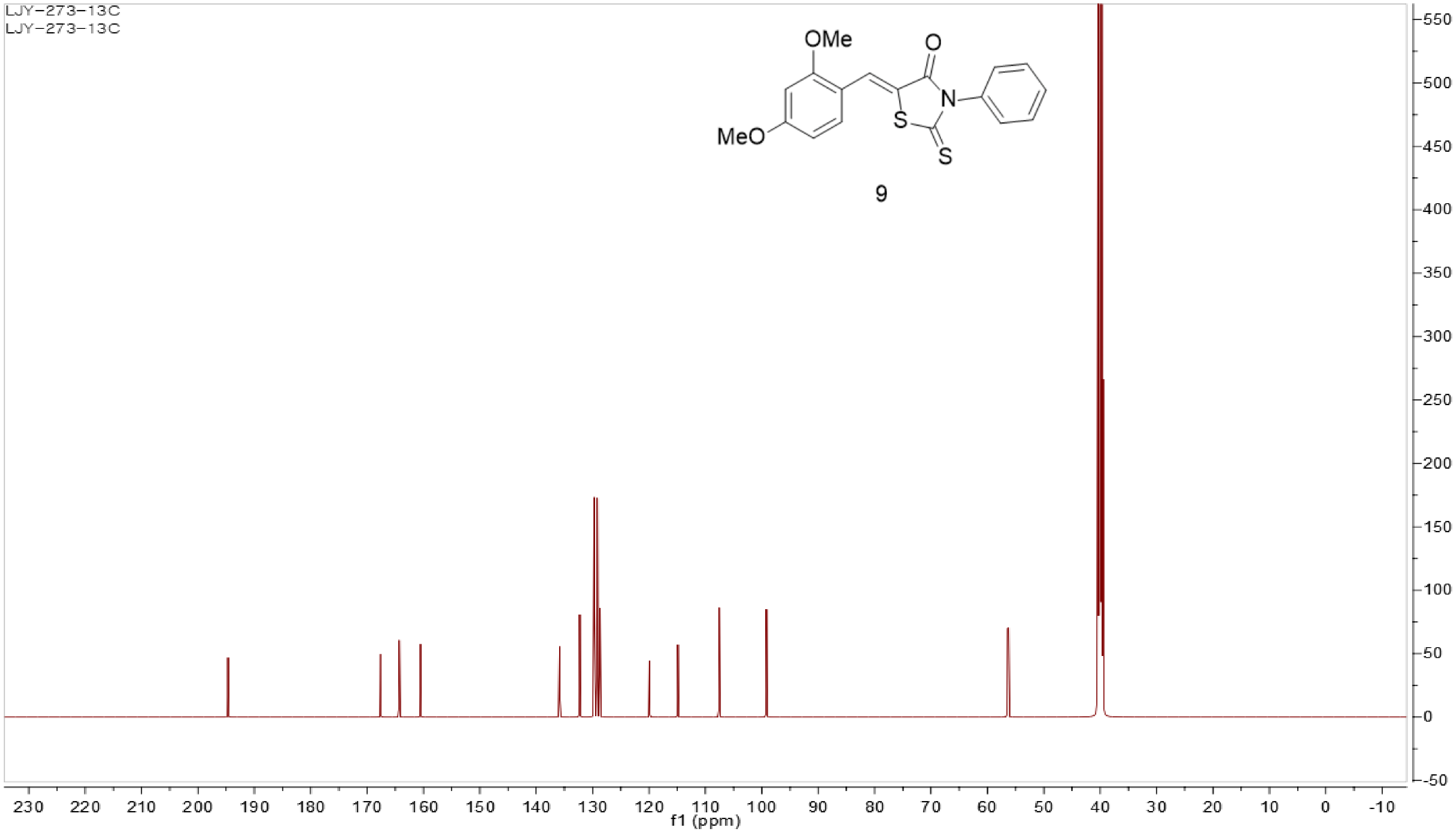

Figure S34. ^{13}C NMR spectrum of compound **9**

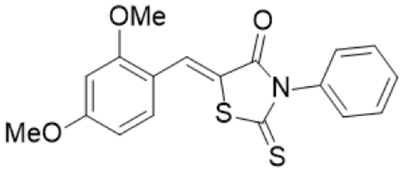

9

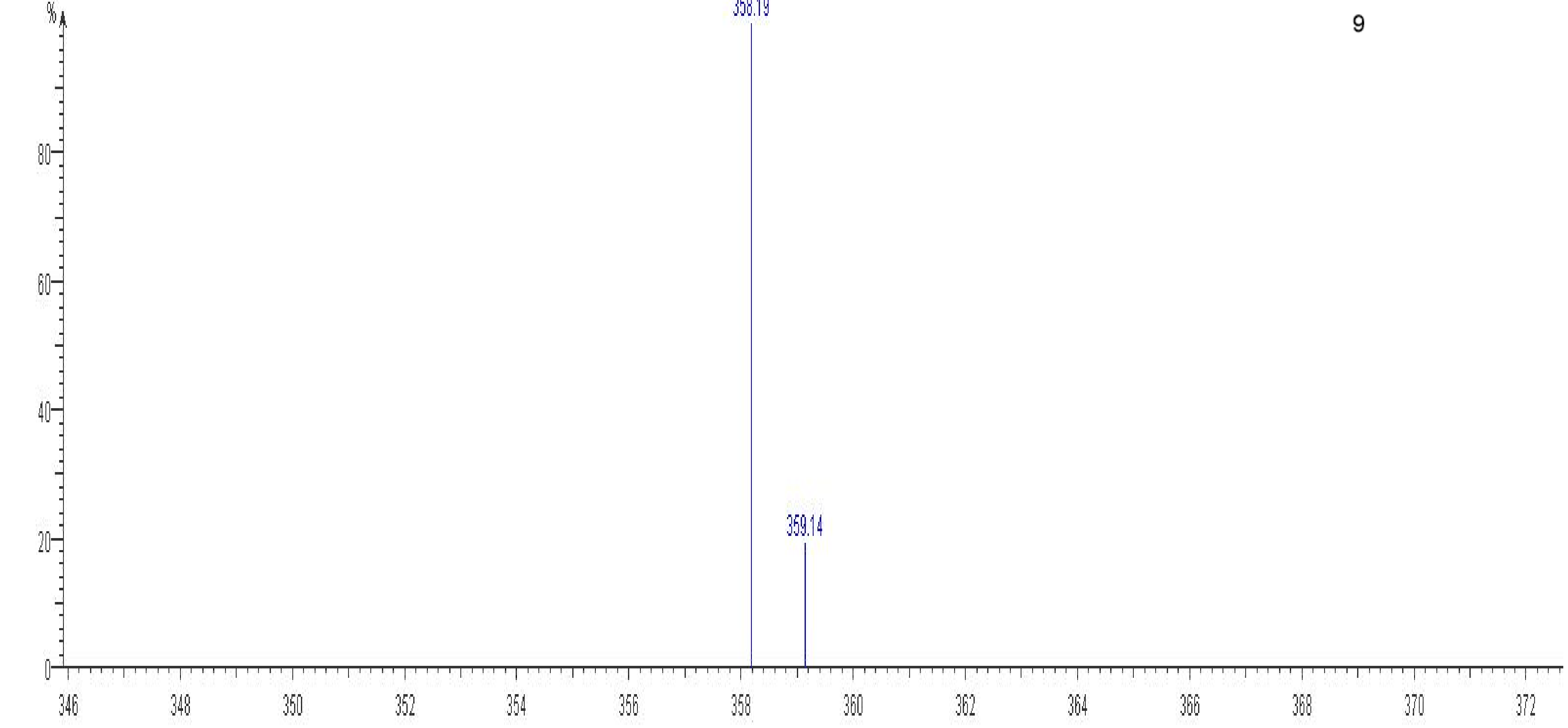

Figure S35. LRMS spectrum of compound **9**

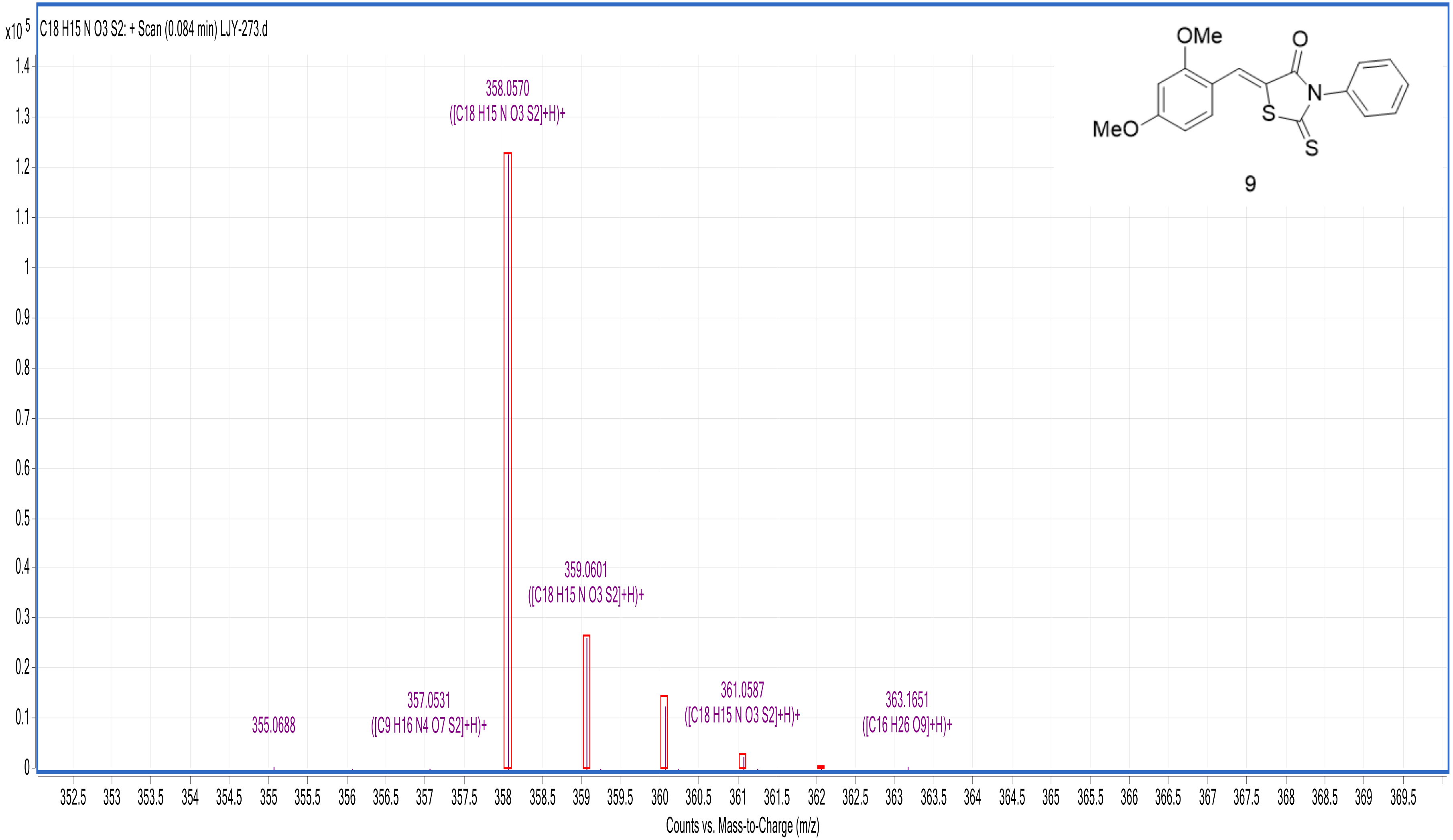

Figure S36. HRMS spectrum of compound **9**

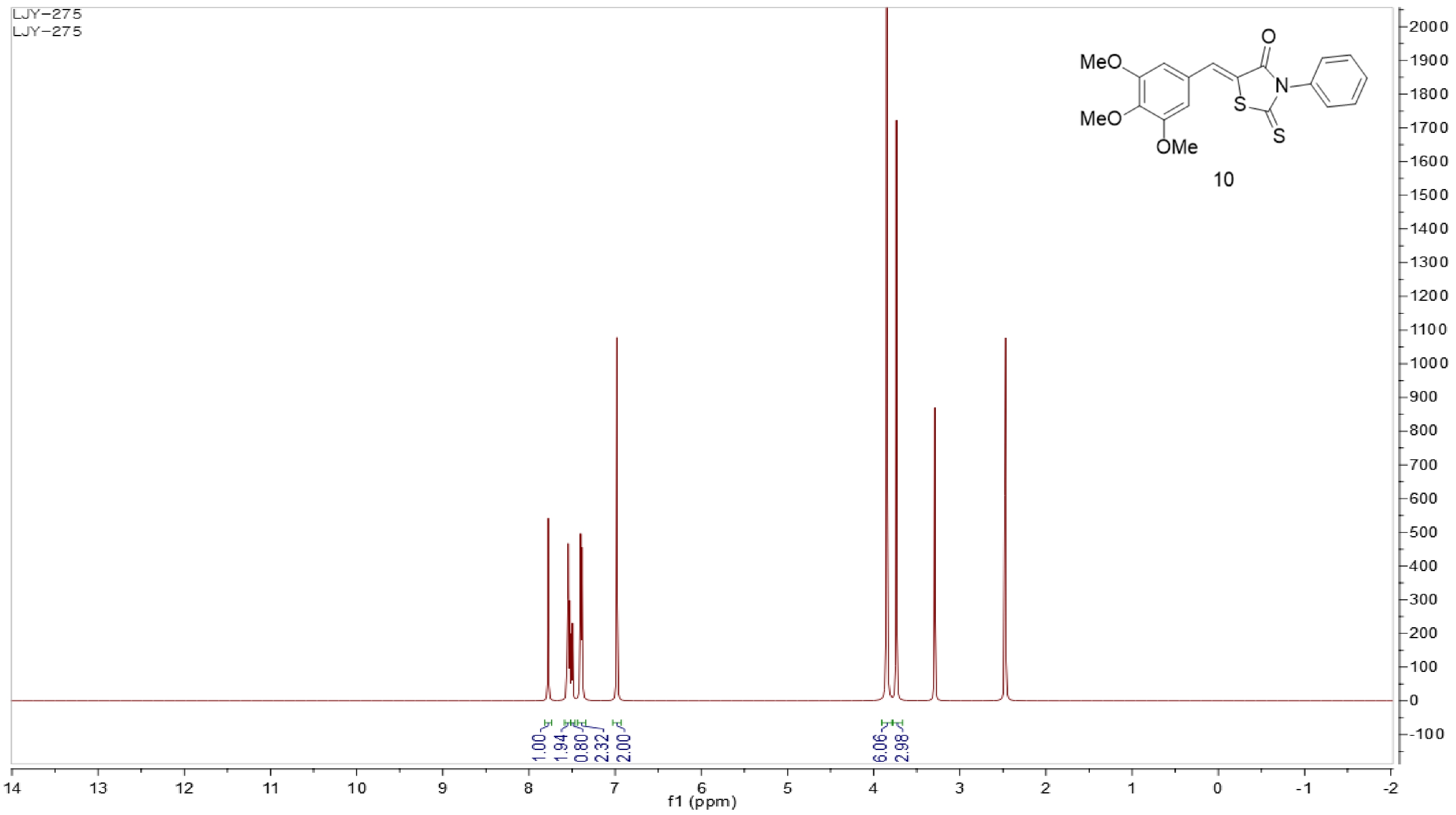

Figure S37. ^{1}H NMR spectrum of compound **10**

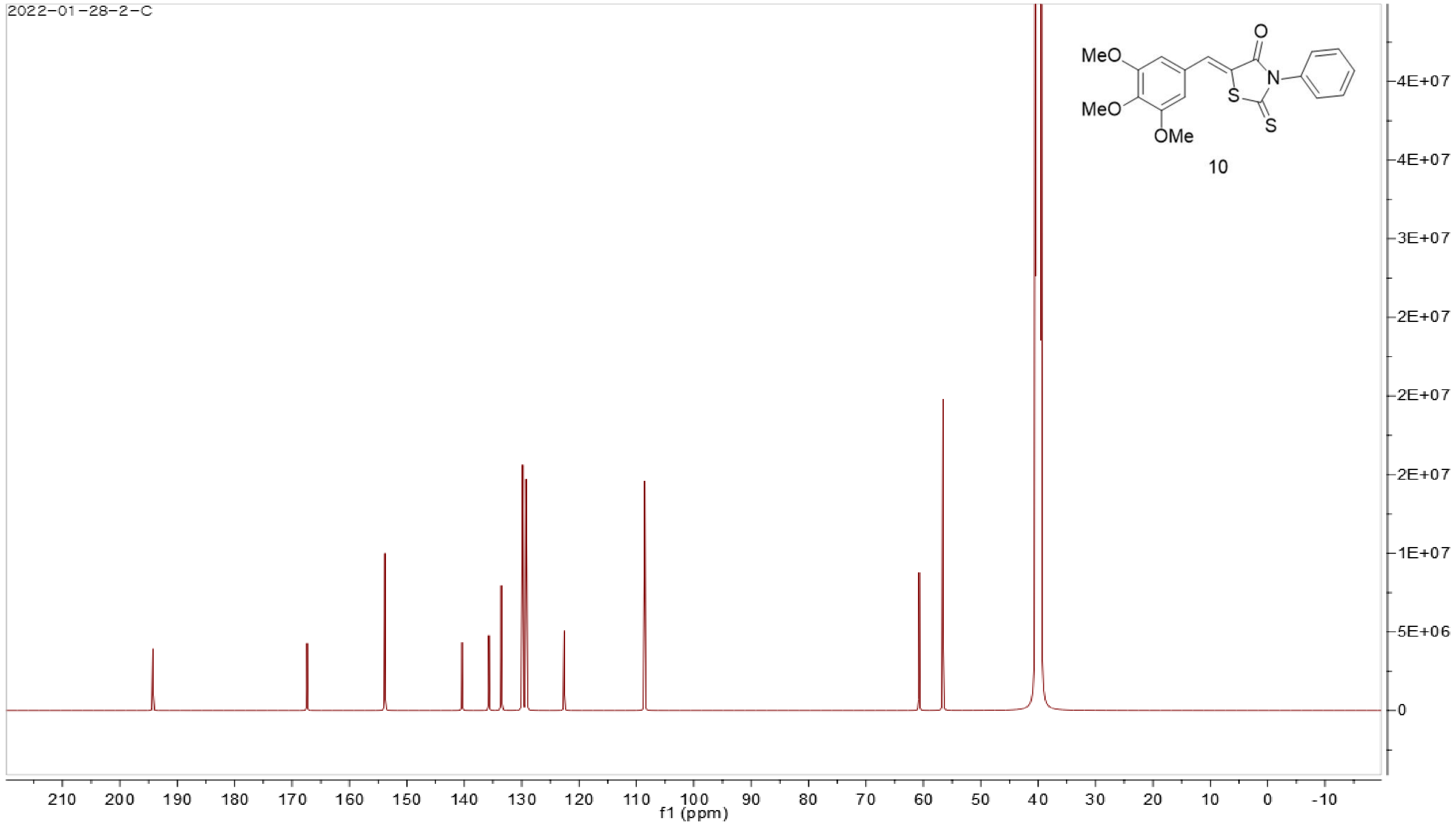

Figure S38. ^{13}C NMR spectrum of compound **10**

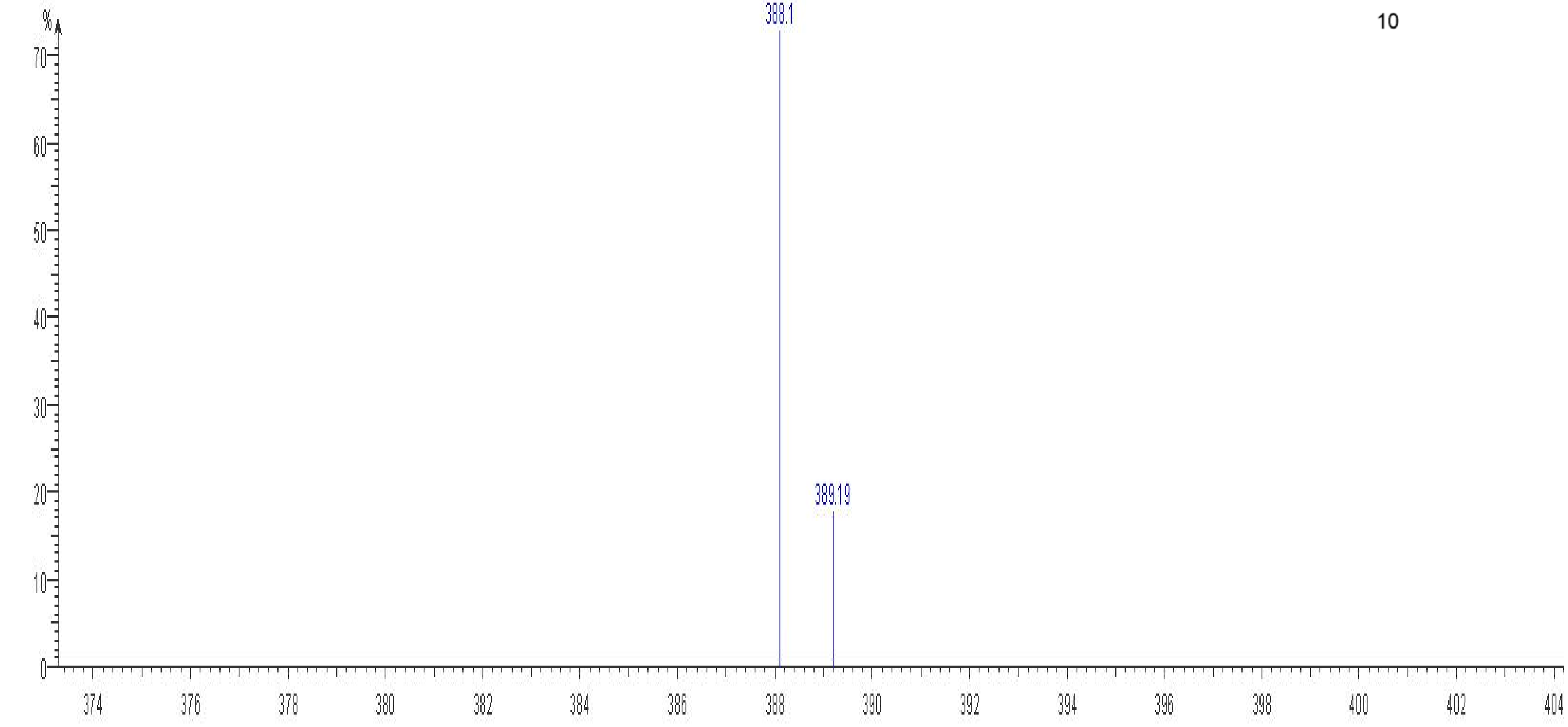

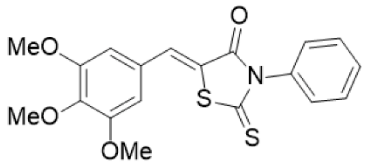

10

Figure S39. LRMS spectrum of compound **10**

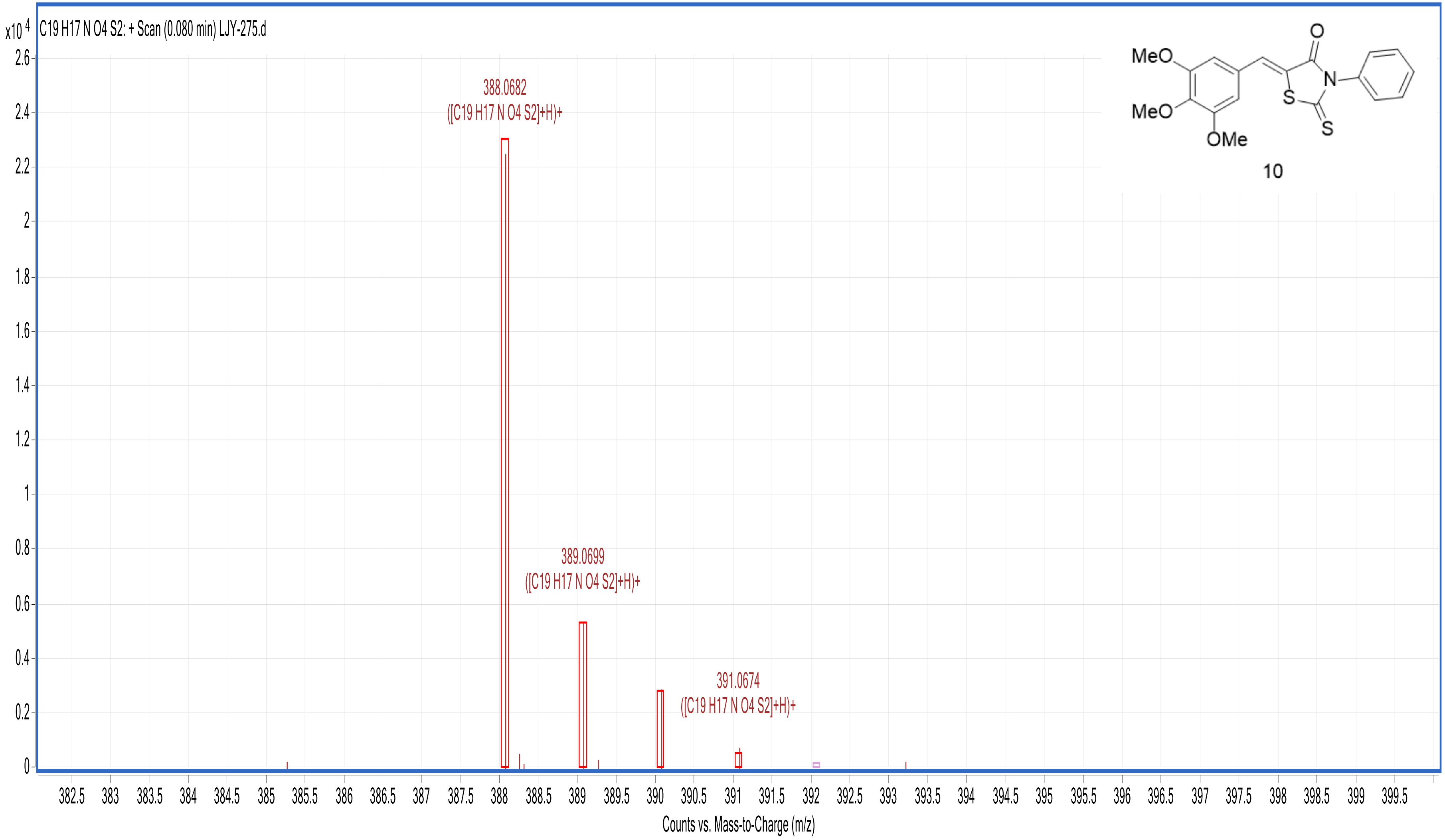

Figure S40. HRMS spectrum of compound **10**

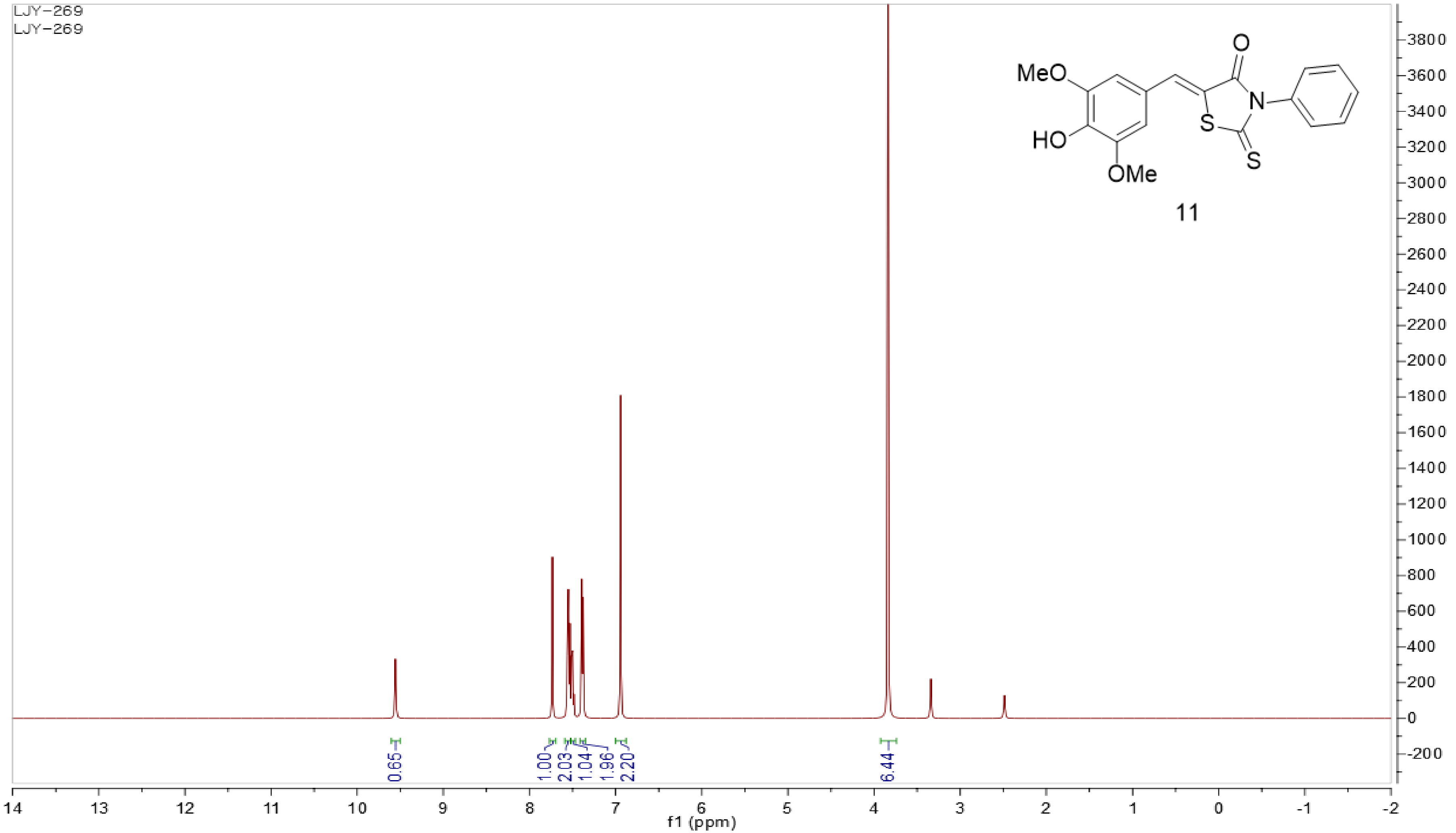

Figure S41. ^{1}H NMR spectrum of compound **11**

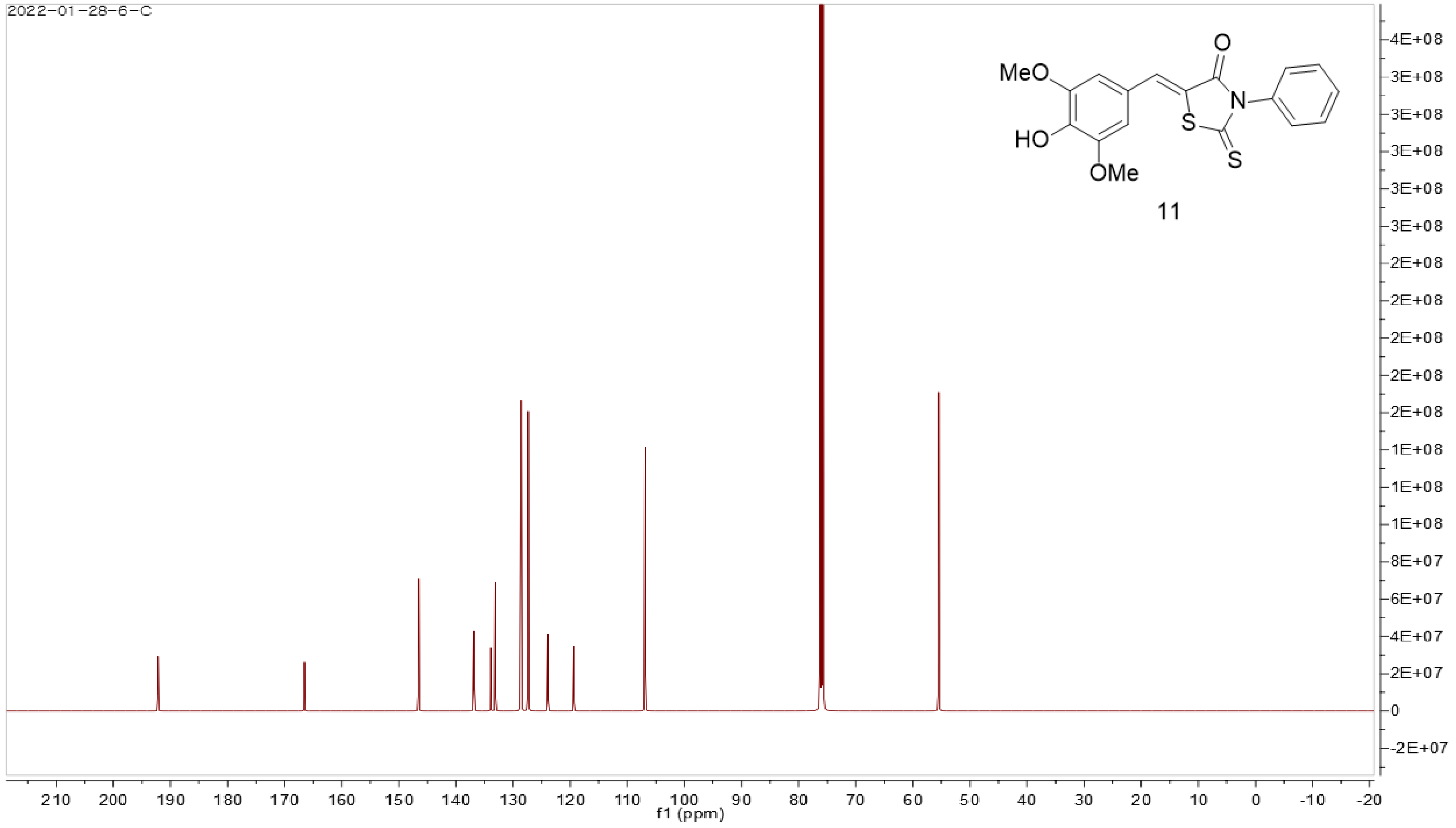

Figure S42. ^{13}C NMR spectrum of compound **11**

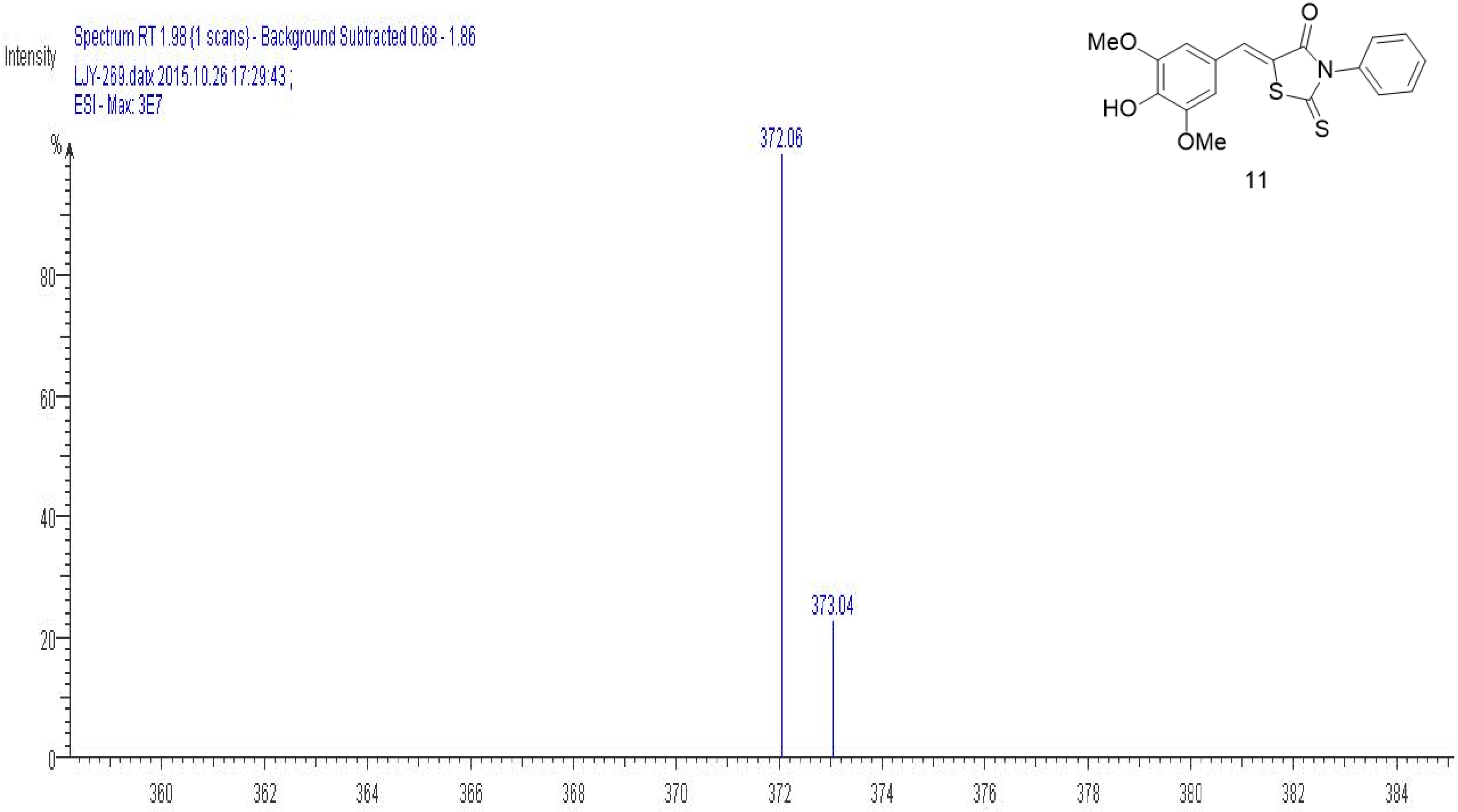

Figure S43. LRMS spectrum of compound **11**

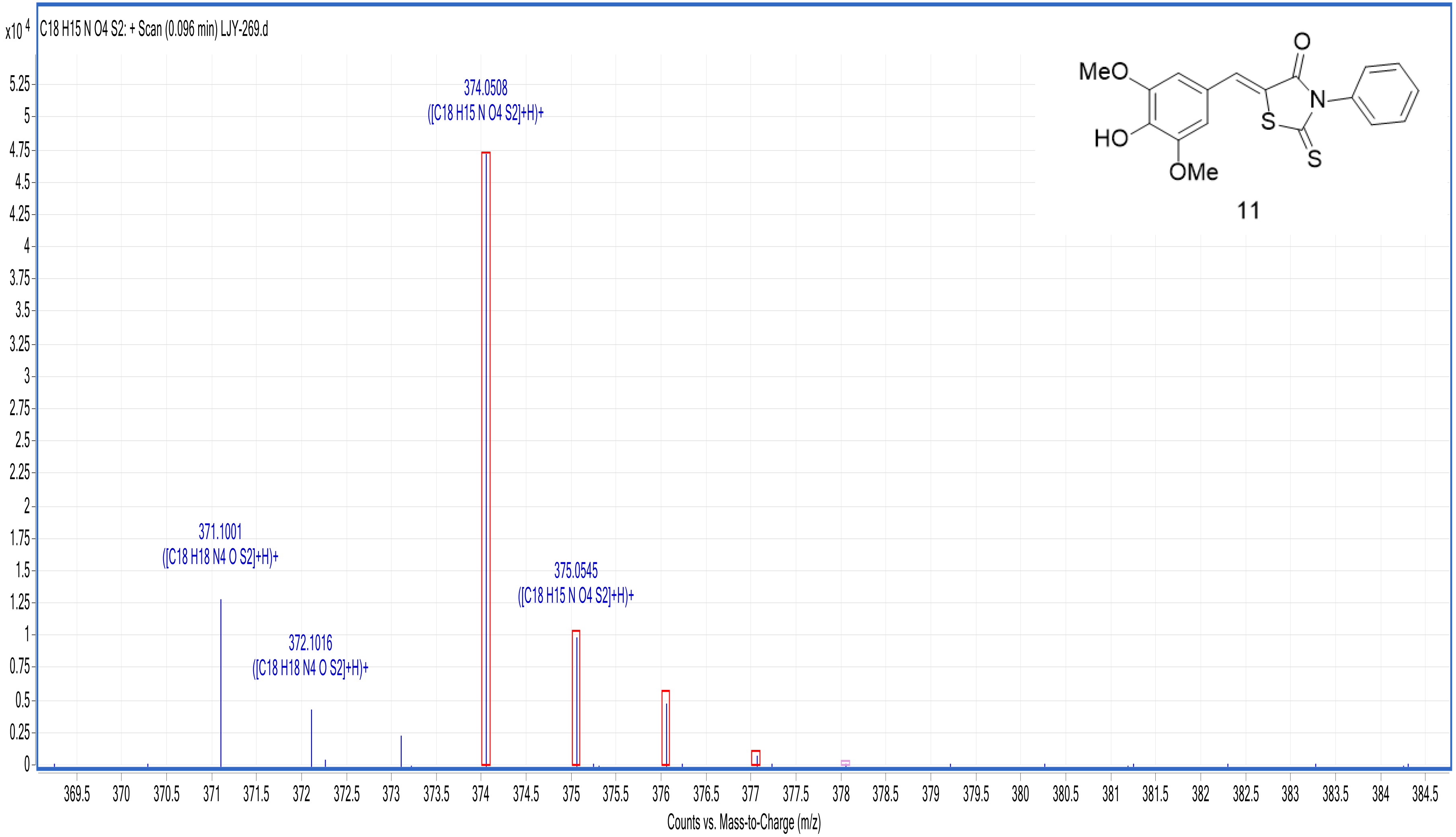

Figure S44. HRMS spectrum of compound **11**

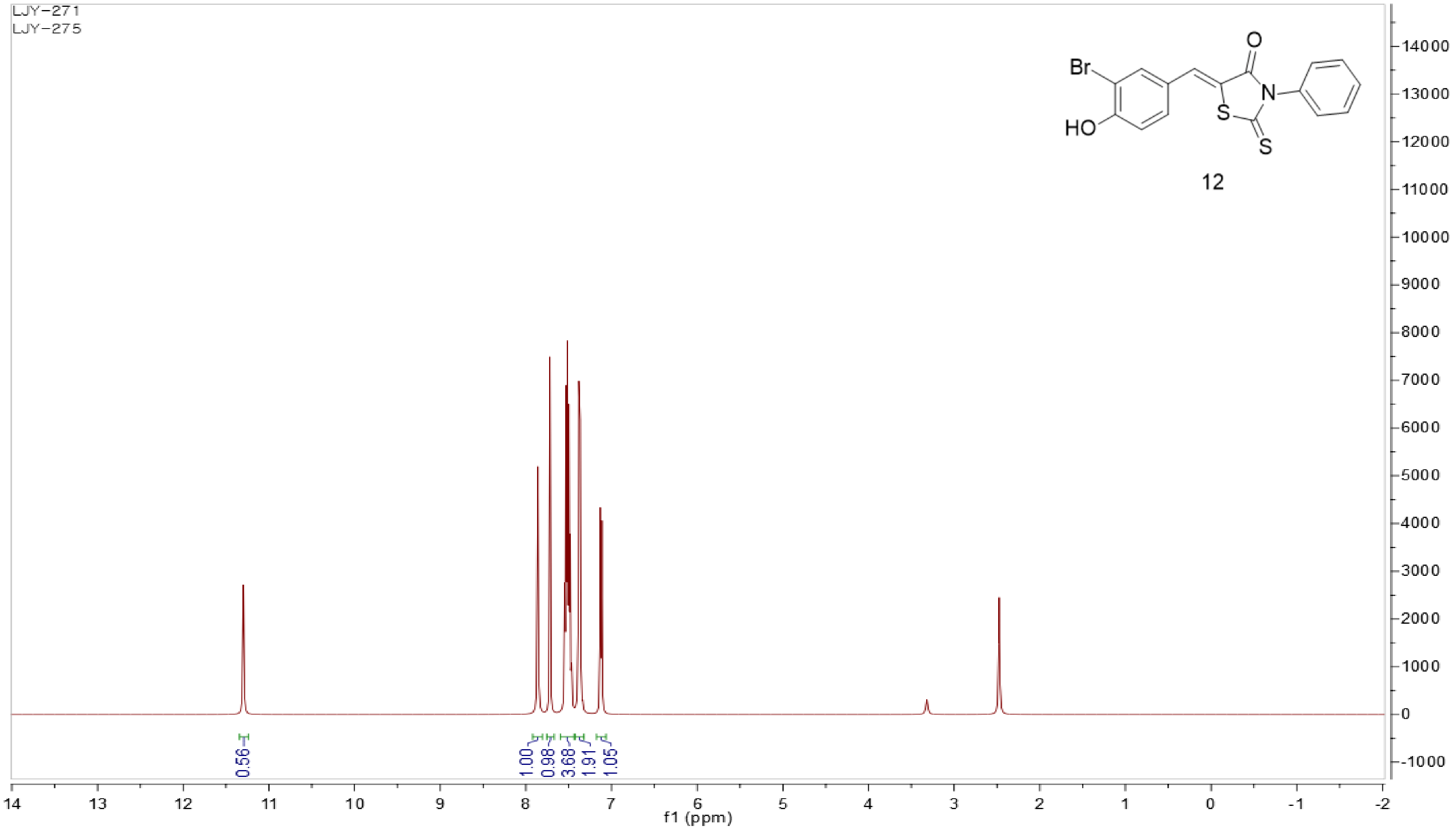

Figure S45. ^{1}H NMR spectrum of compound **12**

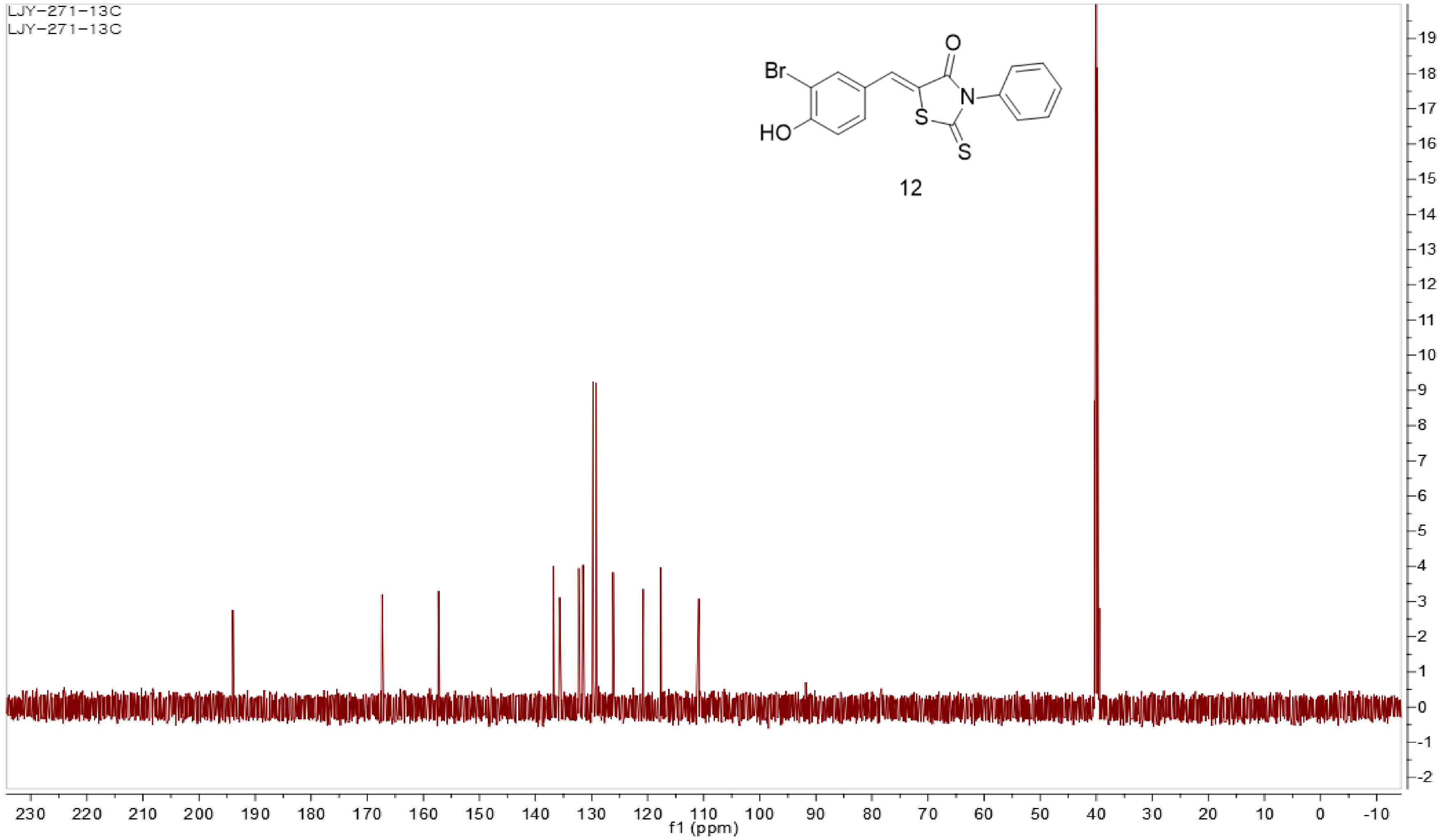

Figure S46. ^{13}C NMR spectrum of compound **12**

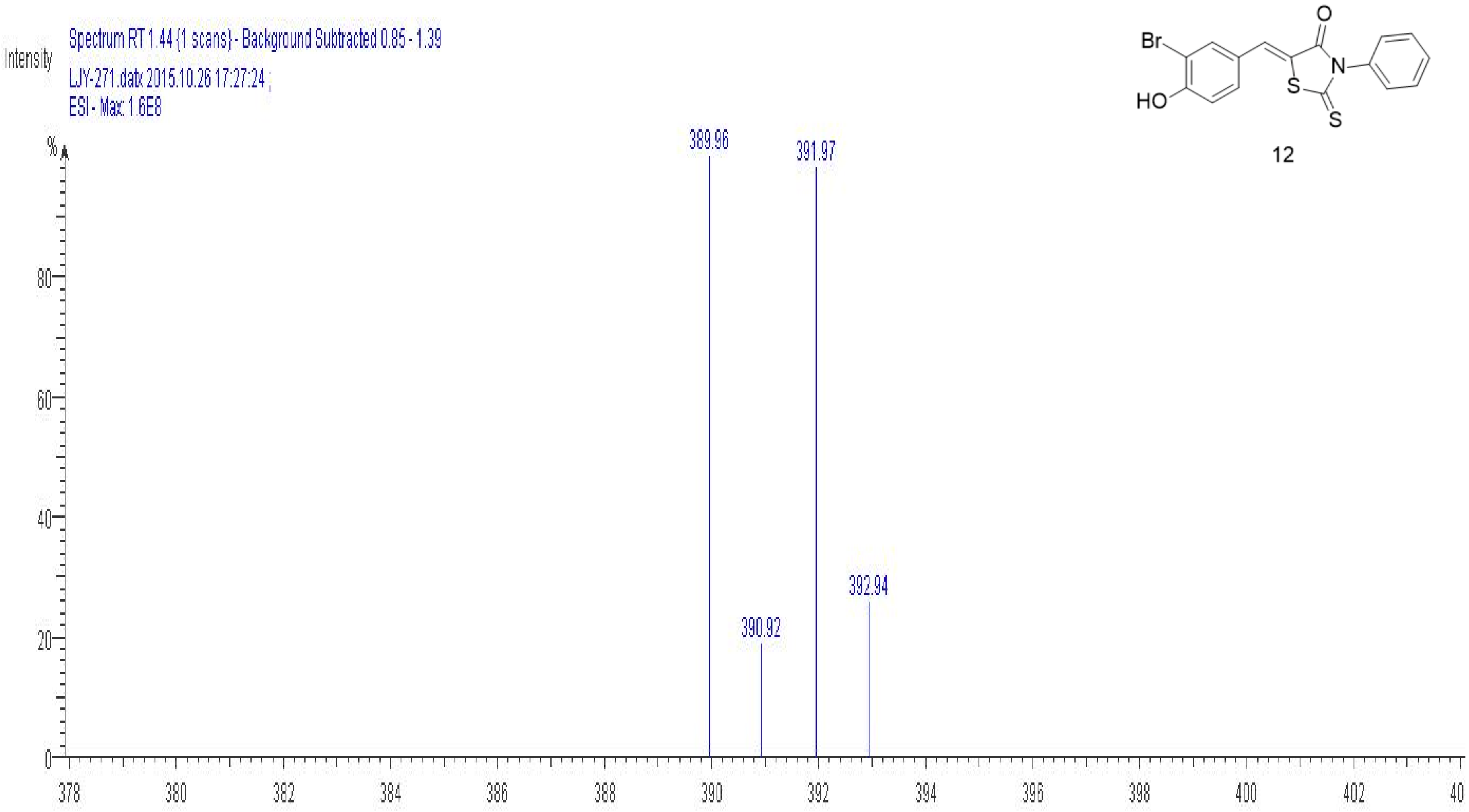

12

Figure S47. LRMS spectrum of compound **12**

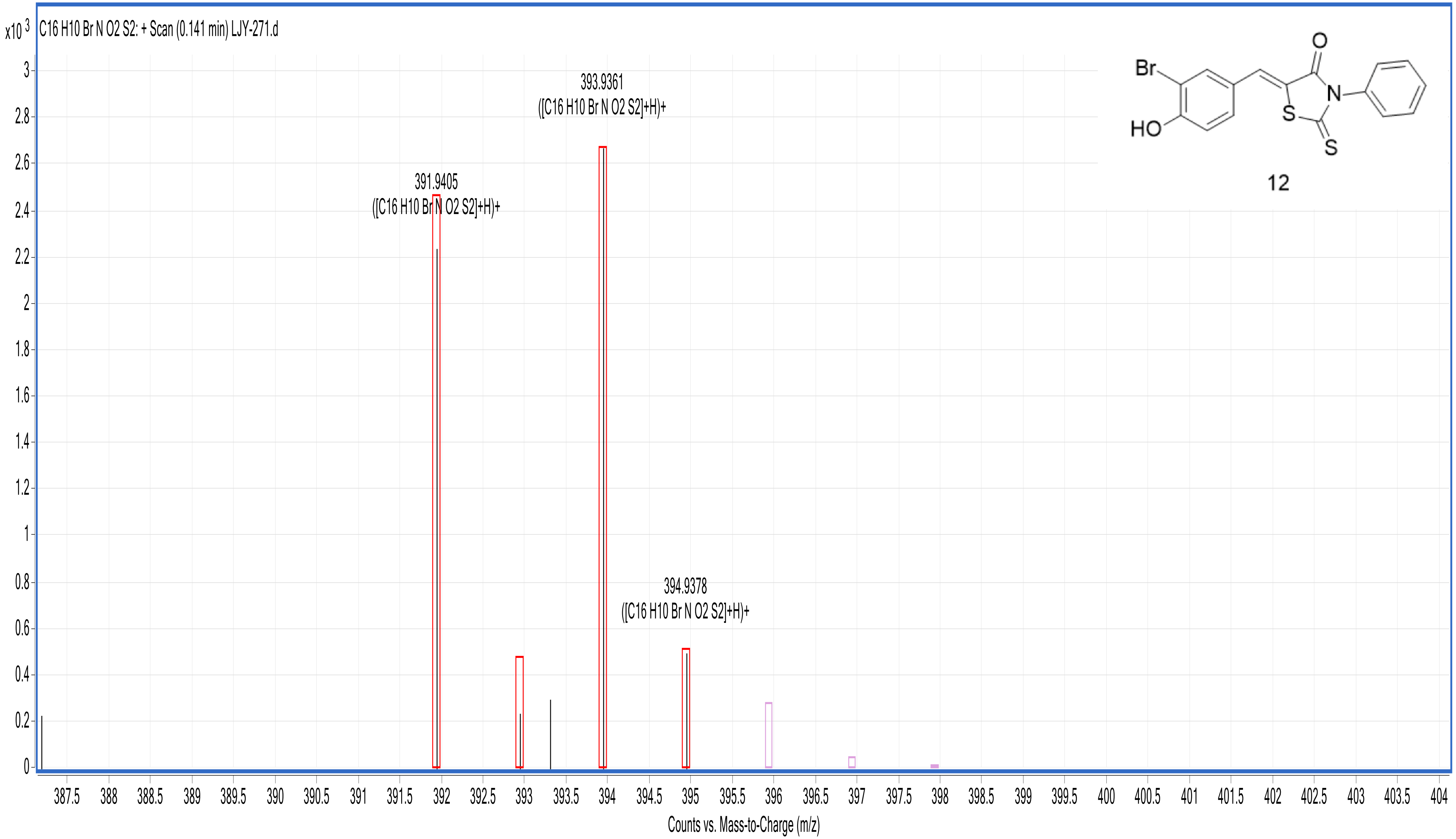

Figure S48. HRMS spectrum of compound **12**

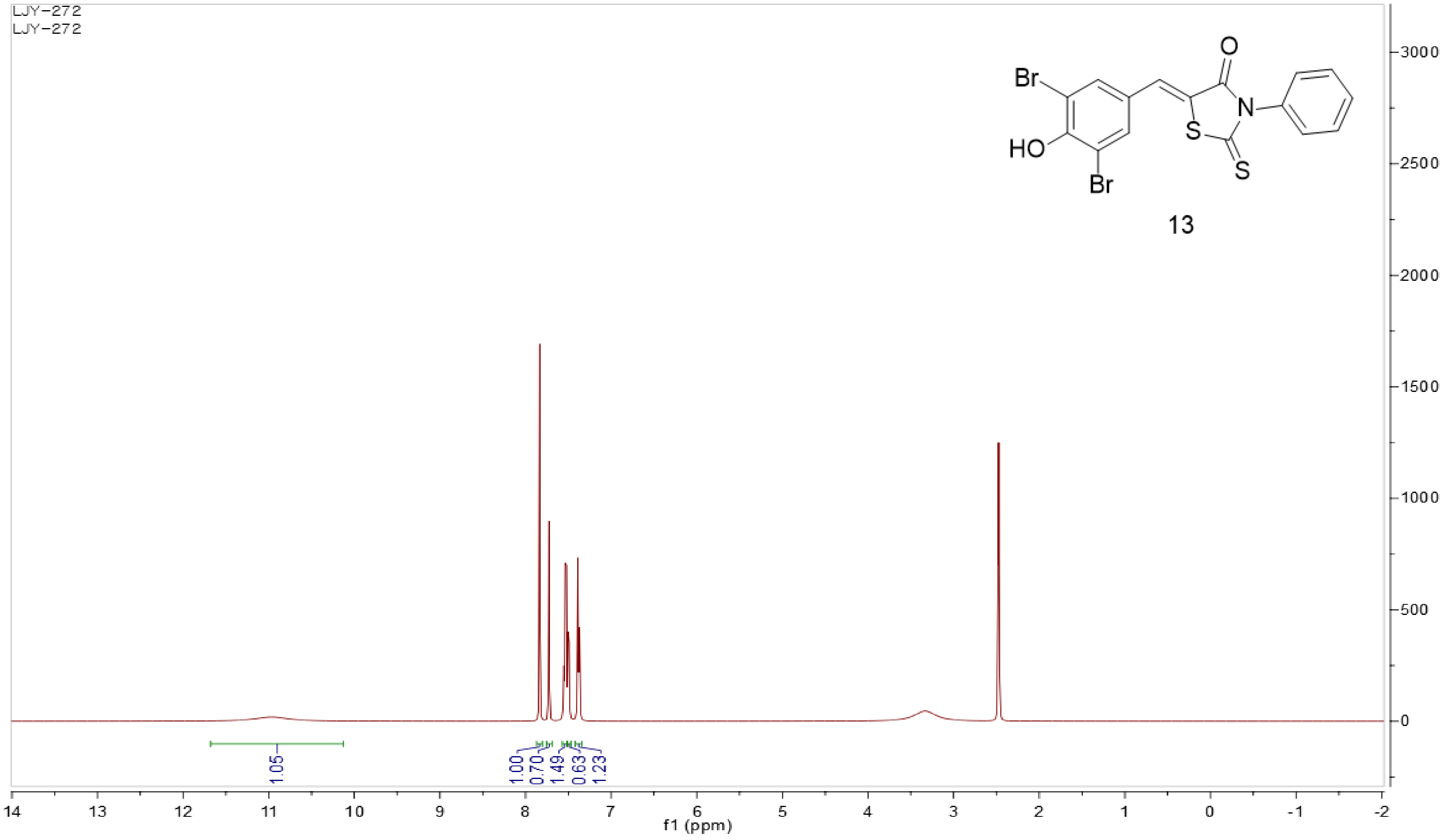

Figure S49. ^{1}H NMR spectrum of compound **13**

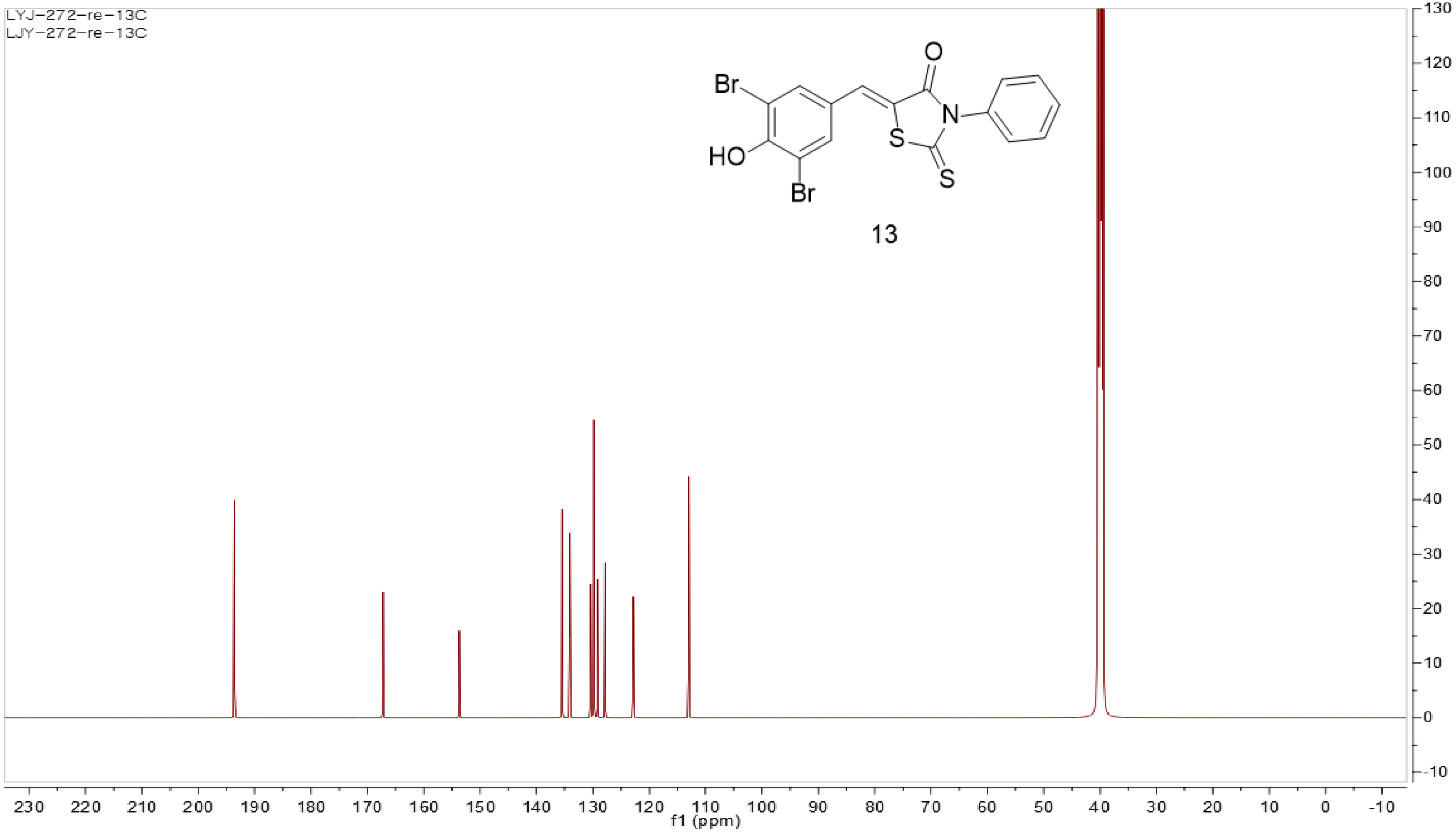

Figure S50. ^{13}C NMR spectrum of compound **13**

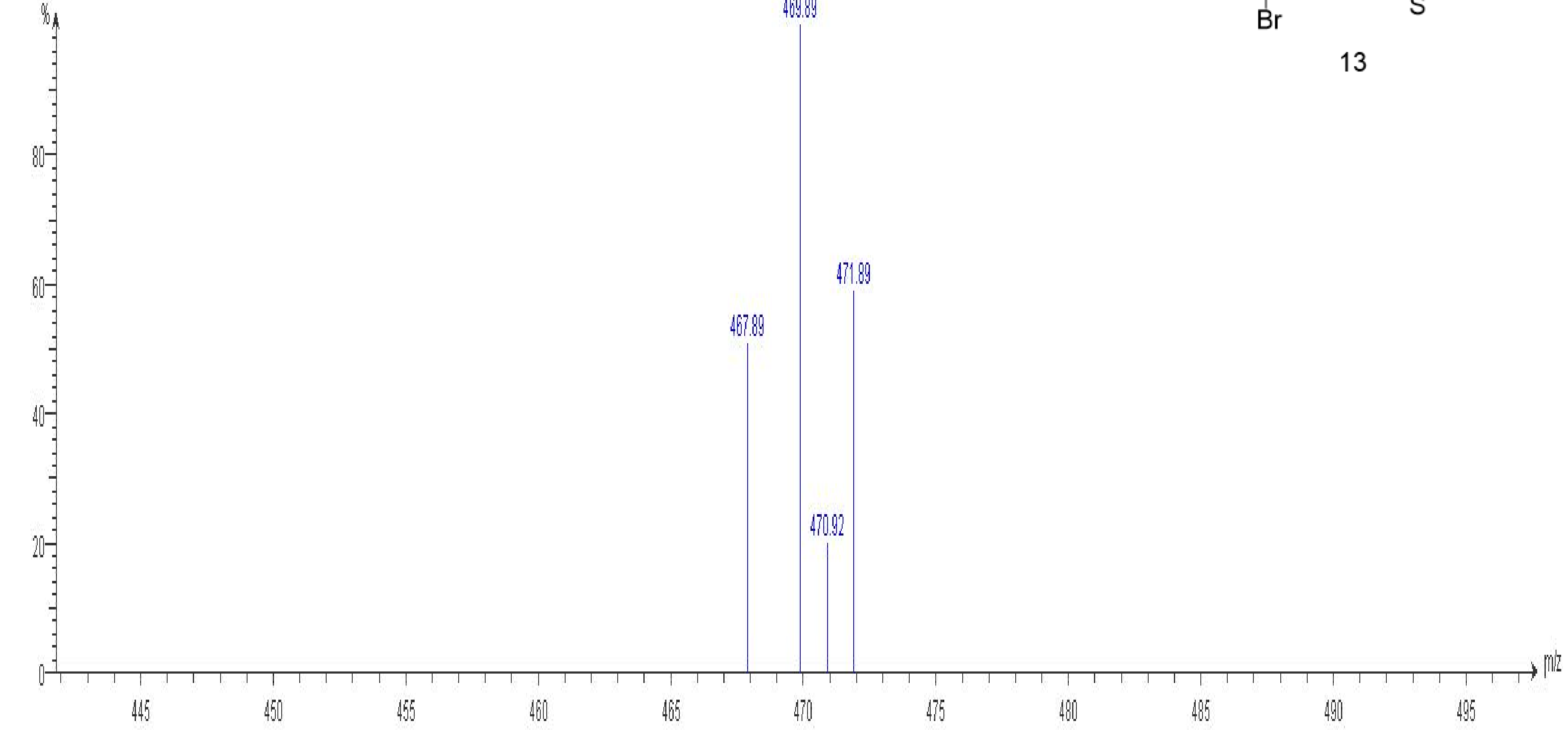

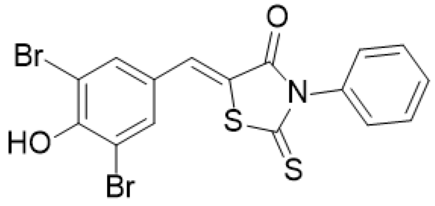

13

Figure S51. LRMS spectrum of compound **13**

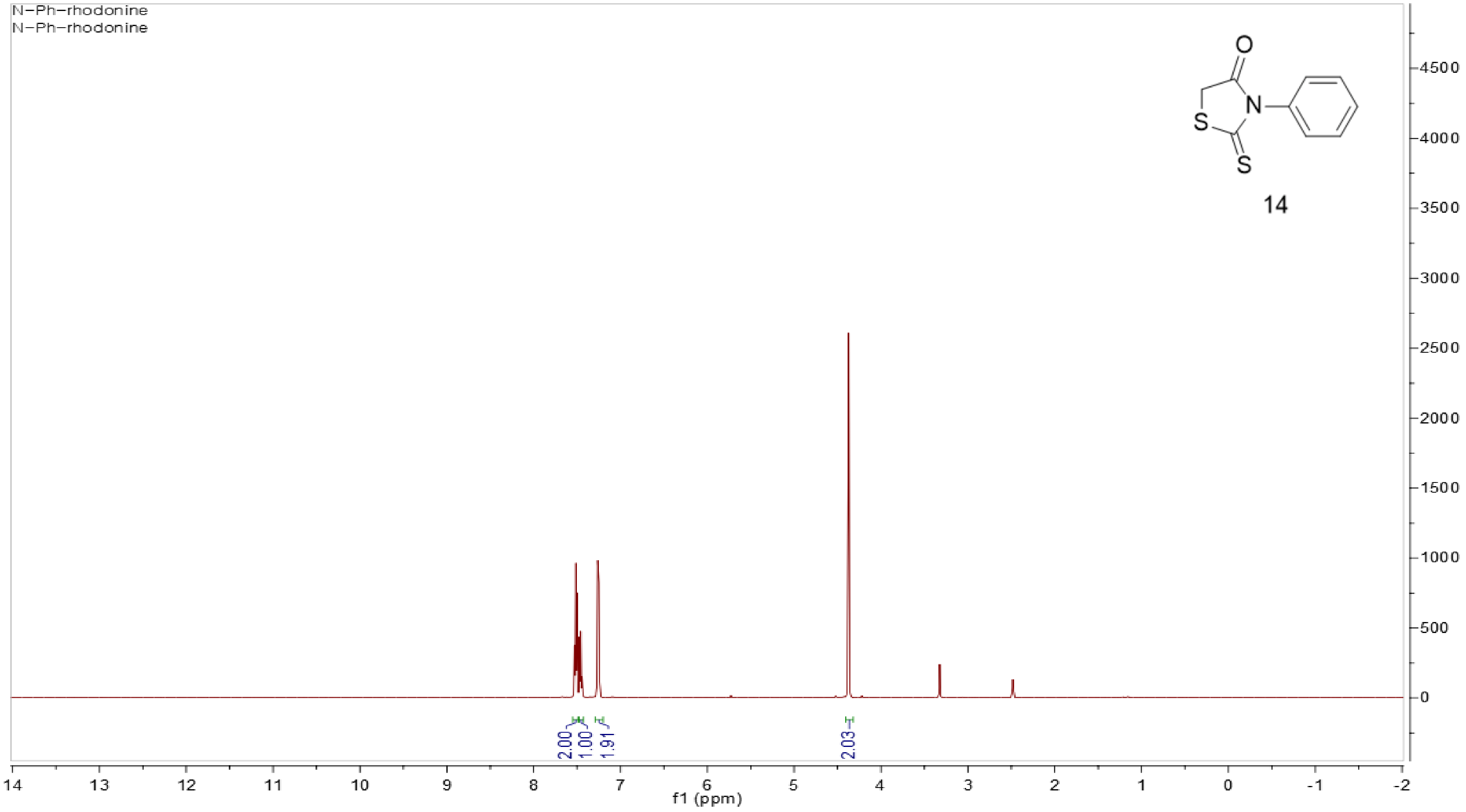

Figure S52. ^{1}H NMR spectrum of compound **14**

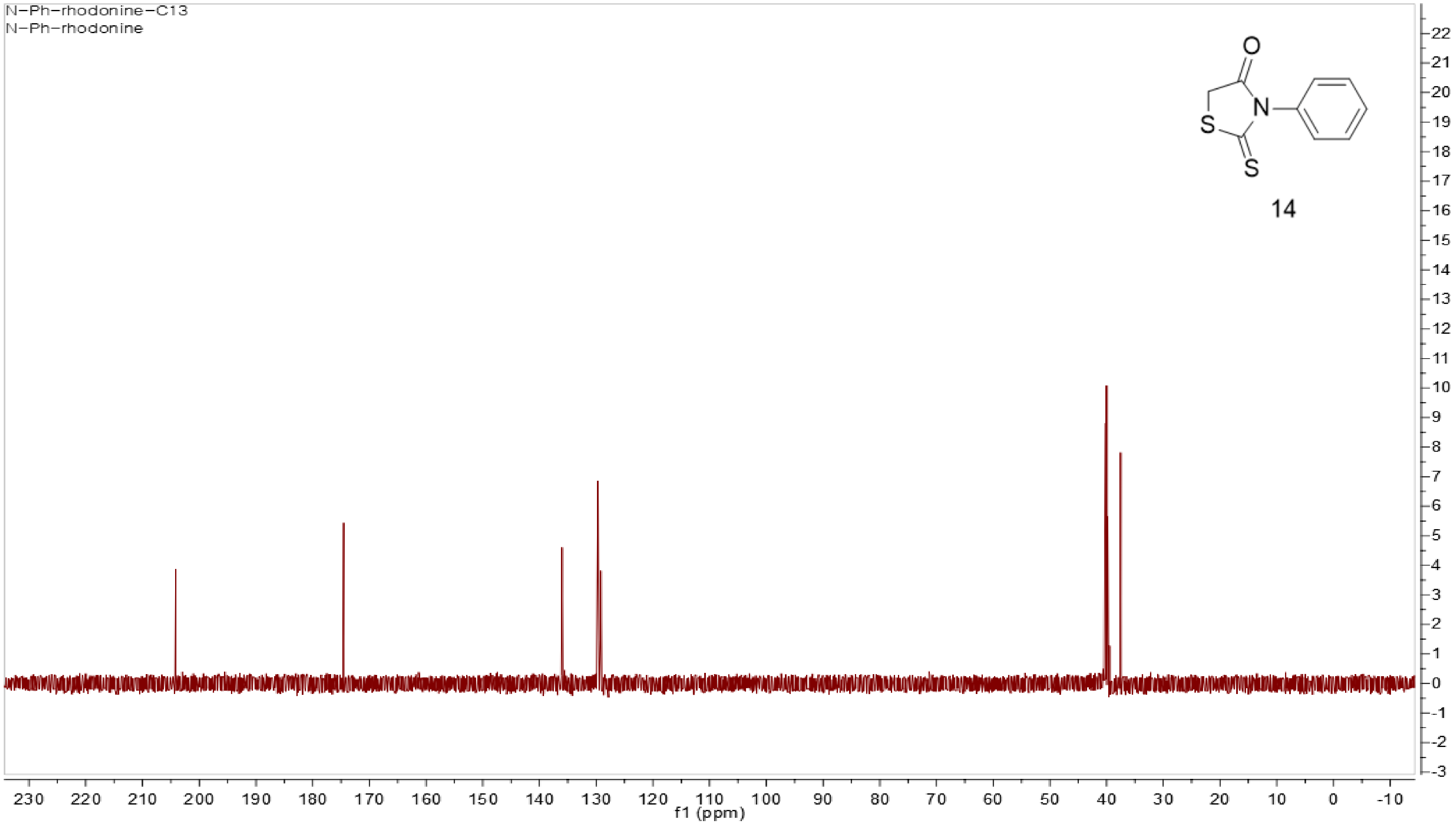

Figure S53. ^{13}C NMR spectrum of compound **14**

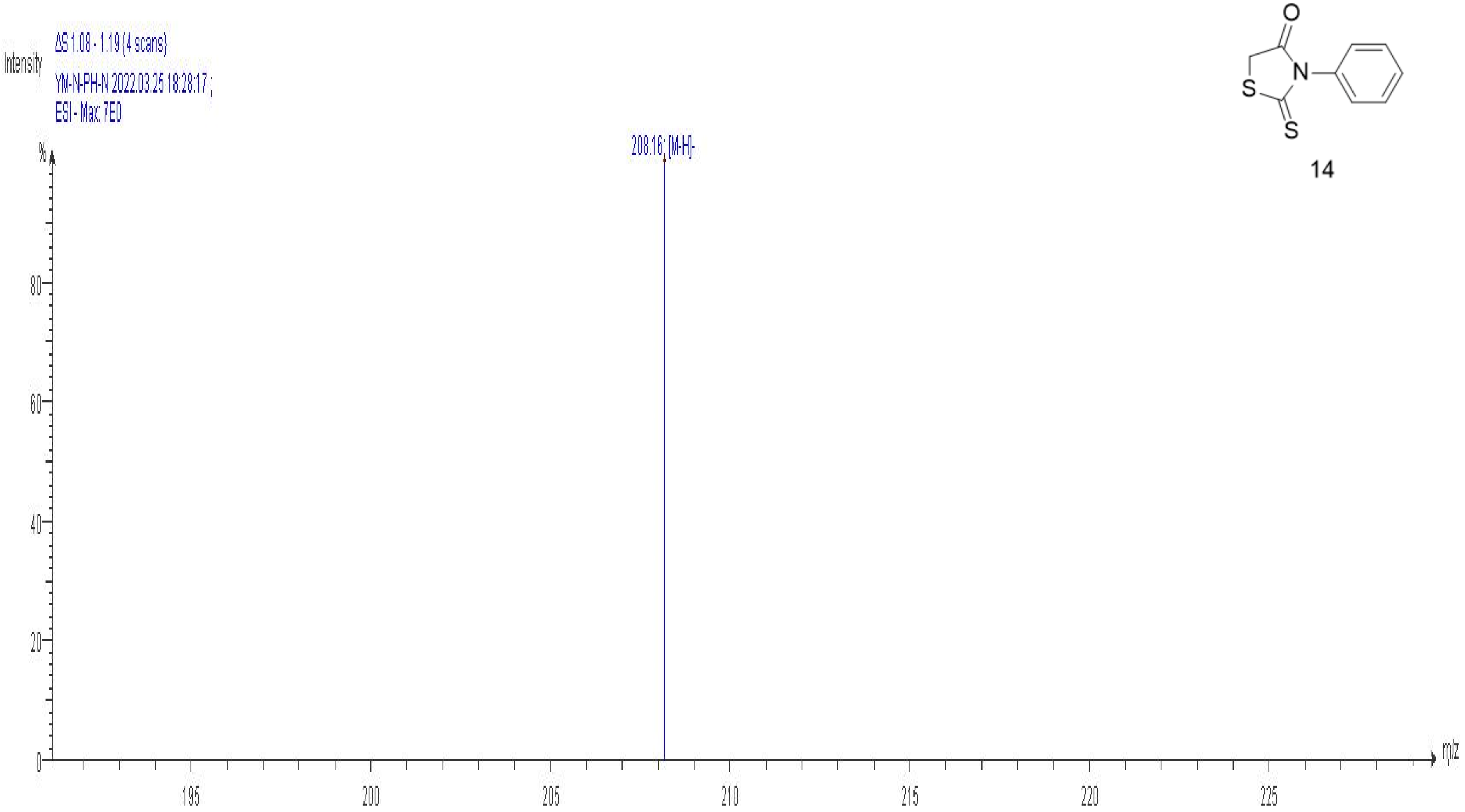

14

Figure S54. LRMS spectrum of compound **14**

Figure S55. Primer sets used for qRT-PCR

Genes	Gene accession number[a]	Ensembl Version[a]	Primers Sequence (Forward: 5'→3', Reverse: 3'→5')
Tyrosinase	NC_ 000073.7	ENSMUSG00000004651.7	Forward = GGAACAGCAACGAGCTAAGG<br>Reverse = TGATGATCCGATTCACCAGA
TRP-1	NC_000070.7	ENSMUSG00000005994.15	Forward = GGAACAGCAACGAGCTAAGG<br>Reverse = TGATGATCCGATTCACCAGA
TRP-2	NC_000080.7	ENSMUSG00000022129.5	Forward = GGAACAGCAACGAGCTAAGG<br>Reverse = TGATGATCCGATTCACCAGA
β-actin	NC_000071.7	ENSMUSG00000029580	Forward = TGTCCACCTTCCAGCAGATGT<br>Reverse = GCTCAGTAACAGTCCGCCTAGA

[a]https://www.ncbi.nlm.nih.gov/gene.
